# Transmembrane Segment XI of the Na^+^/H^+^ Antiporter of *S. pombe* is a Critical Part of the Ion Translocation Pore

**DOI:** 10.1038/s41598-017-12701-z

**Published:** 2017-10-16

**Authors:** Debajyoti Dutta, Kyungsoo Shin, Jan K. Rainey, Larry Fliegel

**Affiliations:** 1 Department of Biochemistry, University of Alberta, Edmonton, Alberta T6G 2H7 Canada; 2 Department of Biochemistry & Molecular Biology, Dalhousie University, Halifax, Nova Scotia B3H 4R2 Canada; 3 Department of Chemistry, Dalhousie University, Halifax, Nova Scotia B3H 4R2 Canada

## Abstract

The Na^+^/H^+^ exchanger of the plasma membrane of *S. pombe* (*Sp*NHE1) removes intracellular sodium in exchange for an extracellular proton. We examined the structure and functional role of amino acids 360–393 of putative transmembrane (TM) segment XI of *Sp*NHE1. Structural analysis suggested that it had a helical propensity over amino acids 360–368, an extended region from 369–378 and was helical over amino acids 379–386. TM XI was sensitive to side chain alterations. Mutation of eight amino acids to alanine resulted in loss of one or both of LiCl or NaCl tolerance when re-introduced into *Sp*NHE1 deficient *S. pombe*. Mutation of seven other amino acids had minor effects. Analysis of structure and functional mutations suggested that Glu^361^ may be involved in cation coordination on the cytoplasmic face of the protein with a negative charge in this position being important. His^367^, Ile^371^ and Gly^372^ were important in function. Ile^371^ may have important hydrophobic interactions with other residues and Gly^372^ may be important in maintaining an extended conformation. Several residues from Val^377^ to Leu^384^ are important in function possibly involved in hydrophobic interactions with other amino acids. We suggest that TM XI forms part of the ion translocation core of this Na^+^/H^+^ exchanger.

## Introduction

Na^+^/H^+^ exchangers are a family of integral membrane proteins that exist in plants, yeast and mammalian cells^1^. In mammalian cells, they function to remove excess intracellular protons in exchange for external sodium and are important in the pathology of several diseases including ischemic heart disease and breast cancer^2,3^. In plants and yeast, plasma membrane members of this family of proteins also catalyze the exchange of sodium for protons, but to serve a different end. They function in salt tolerance. Excess intracellular sodium is toxic and one way in which yeast and plants deal with sodium load is to extrude the ion through these plasma membrane transporters. In plants, different types of plasma membrane Na^+^/H^+^ antiporters remove sodium. The energy of transport comes from a proton gradient that is generated by the plasma membrane H^+^-ATPase^4^. Overexpression of plasma membrane salt tolerance proteins can improve salt tolerance in plants^5^.

In yeast, plasma membrane Na^+^/H^+^ antiporters also serve to mediate salt tolerance by removal of intracellular sodium in exchange for extracellular protons. The fission yeast *Schizosaccharomyces pombe* is an excellent system in which to study plasma membrane salt tolerant proteins. In *S. pombe*, the Na^+^/H^+^ exchanger *Sp*NHE1 (previously known as sod2) plays the major role in salt removal from the cytosol and in salt tolerance. NHE1 of *S. pombe* can convey salt tolerance to plants and can function at the plasma membrane^6^ and *Sp*NHE1 clusters with plant plasma membrane salt tolerance proteins in phylogenetic analysis^7^. Also, there are limited other salt tolerance mechanisms in *S. pombe* so the disruption of *Sp*NHE1 yields a salt sensitive phenotype^8^. This makes *Sp*NHE1 in *S. pombe* an ideal system for characterizing the protein’s activity and we have earlier^9,10^ used this system to study the effect of specific mutations of *Sp*NHE1 on transport.

Mechanisms of transport of plasma membrane Na^+^/H^+^ exchangers and of ion transporting proteins in general, are an important fundamental scientific problem. The structures of a few Na^+^/H^+^ exchangers such the *E. coli* sodium transporter NhaA^11^ and those of *Thermos thermophilus* (NapA)^12^, *Methanococcus jannaschii* (MjNhaP1) *Pyrococcus abyssi* (PaNhaP)^13^ have been characterized, but most, including humans and those of higher eukaryotes, are not well studied. In the well studied Na^+^/H^+^ exchangers, one key feature that has been noted is the critical nature of transmembrane (TM) segments IV and XI. These two segments create a characteristic fold. Both are discontinuous helices and at their crossing point are unfolded, accommodating charged and polar residues that neutralize the dipoles of the helices within the lipid bilayer. This region may harbor the ion translocation center^11,14^. This fascinating arrangement and the details of these transmembrane segments have not been well demonstrated in other Na^+^/H^+^ exchangers of higher species such as in vertebrate, plant or yeast Na^+^/H^+^ exchangers.

In this study, we examined the structure and function of TM XI of *Sp*NHE1. We used alanine scanning mutagenesis to characterize functional residues of this region of the protein. Additionally, we examined the structure of this TM segment by NMR spectroscopy. This region appears similarly structured to other Na^+^/H^+^ exchangers, with a putative helix-extended region-helix in dodecylphosphocholine (DPC) micelles. A number of regions of the TM segment are important in function, possibly contributing to cation coordination or maintaining structure of this TM segment or in interaction with other transmembrane segments. Our results are the first examination of the structure and function of this region of the membrane protein. They support the hypothesis that *Sp*NHE1 has a “Na^+^/H^+^ exchanger”-like fold within the protein and that amino acids 360–393 are a critical part of this fold.

## Results

### *Sp*NHE1 alignment and modeling

**Figure 1:**
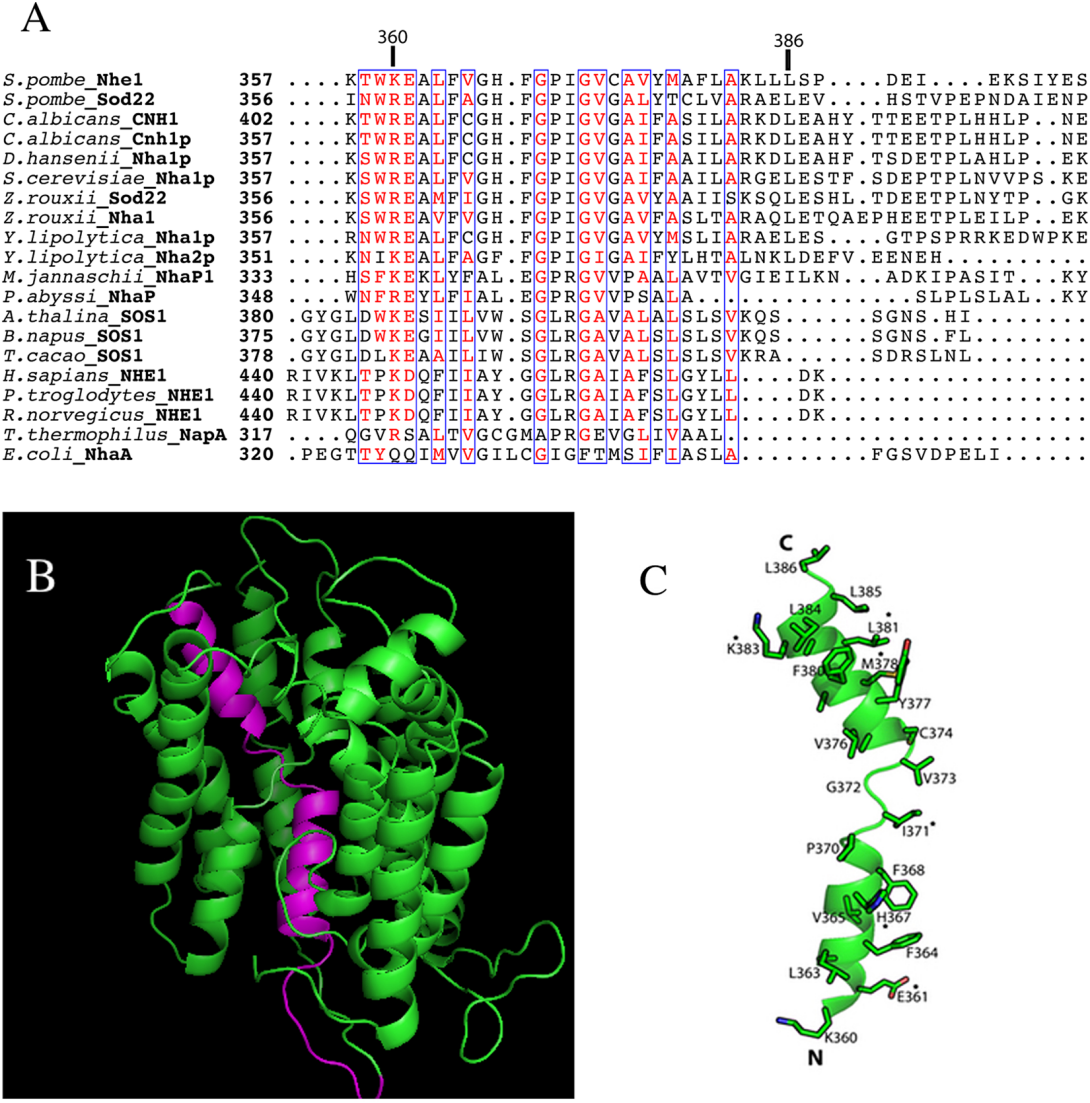
Alignment and molecular modeling of TM XI of *Sp*NHE1. (**A**) Alignment of sequences of yeast, fungi, plant, mammalian and bacterial plasma membrane Na^+^/H^+^ exchangers. Conserved amino acids are colored red and conserved regions are boxed. Representative included are; yeast and fungi group *S*. *pombe* NHE1 (**NP_592782.1**), *S*. *pombe* Sod22 (**NP_594194.1**), *Candida albicans* CNH1 (**XP_710352.1**), *C*. *albicans* Cnh1p (**AAL24468.1**), *Debaryomyces hansenii* Nha1p (**CAI45290.1**), *Saccharomyces cerevisiae* Nha1p (**NP_013239.1**), *Zygosaccharomyces rouxii* Sod22 (**XP_002497045.1**), *Z*. *rouxii* Nha1 (**XP_002497801.1**), *Yarrowia lipolytica* Nha1p (**XP_501299.1**), *Y*. *lipolytica* Nha2p (**XP_503447.1**); *Methanocaldococcus jannaschii* NhaP1 (**NP_247021.1**), *Pyrococcus Abyssii* NhaP (**CAB50204**), *Thermus thermophiles* NapA (**YP_144738**), and *Escherichia coli* NhaA (**WP_000681354**) are included as acheal and bacterial members. From plant groups *Arabidopsis thaliana* SOS1 (**AAL32824**), *Brassica napus* SOS1 (**AGA37213.1**), and *Theobroma cacao* SOS1 (**XP_007045406.1**) are included. Finally, from mammals, *Homo sapiens* NHE1 (**NP_003038.2**), *Pan troglodytes* NHE1 (**XP_016812591**) and *Rattus norvegicus* NHE1 (**AAA98479**) are included. See supplementary Figure 1 for full alignment. (**B**) Molecular model of *Sp*NHE1. The model of *Sp*NHE1^15^ is shown in green. Amino acids 360–393 are shown in purple. Amino acids 169–188 were removed to allow visualization of TM XI. (**C**) Amino acids 360–393 of *Sp*NHE1 model. Asterisk indicates residues found to be important in the present study (see below).

We examined amino acids of the putative TM segment XI of *Sp*NHE1, which we hypothesized is important in activity *S. pombe*. Figure 1A is an alignment of the putative TM XI region of *Sp*NHE1 with several other members of the Na^+^/H^+^ exchanger family of proteins. Supplementary Fig. 1 illustrates the entire alignment of the protein. Multiple members of the family were included. Boxed and red amino acids highlight regions of conservation. There was little conservation beyond amino acid 383 of *Sp*NHE1. This was a general trend in the protein, with regions intervening between transmembrane segments showing little conservation except in some more closely related species (Supplementary Fig. 1). Figure 1B illustrates the putative structure of *Sp*NHE1, which is based on molecular modeling of the protein described earlier^15^. A helical structure was predicted for residues 360–368 and residues 374–385, while an extended region was predicted between these two helical segments (Fig. 1B,C). According to the previous model of *Sp*NHE1, Leu^386^ to Ser^403^ form an extended extracellular loop linking to putative TMXII. There was little conservation of this region of *Sp*NHE1 with other family members.

### NMR Resonance Assignment and Analysis

Well-resolved homonuclear ^1^H-^1^H TOCSY and NOESY experiments and a natural abundance ^1^H-^13^C HSQC experiment were acquired in DPC micelles for a synthetic TM XI peptide. On the basis of these data, we were able to unambiguously sequentially assign all residues (chemical shifts are deposited in the BMRB, accession # 27092) Beyond the exhibition of appropriate spin-system properties for a given amino acid type, these assignments were developed on the basis of unambiguous sequential (i to i+1) and medium range (i to i+2 through i+4) NOE contacts (Supplementary Fig. 2).

**Figure 2:**
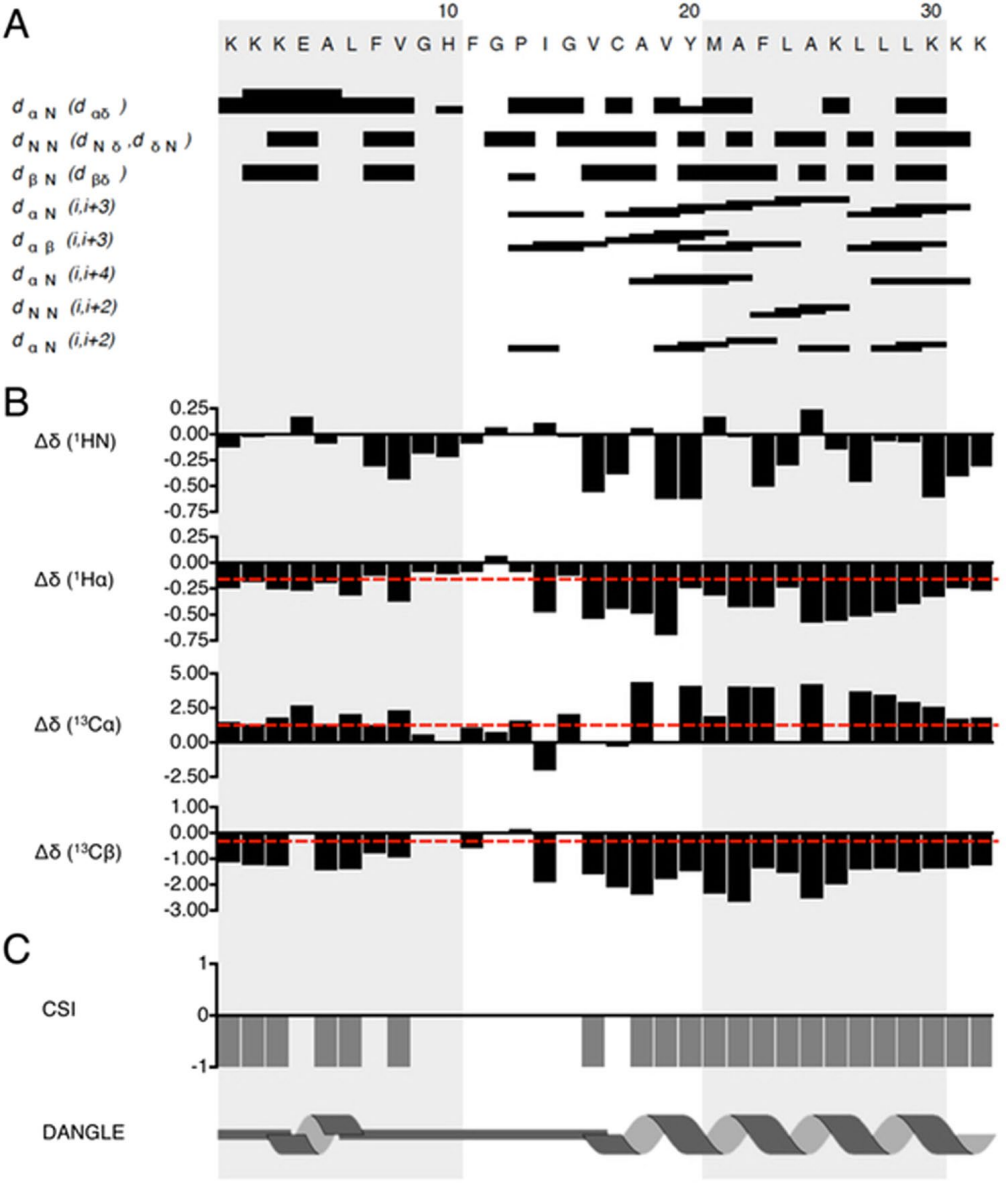
^1^H homonuclear NOE restraint assignments for TM XI in DPC micelles. (**A**) Graphical summary of *d*_*xx*_ NOE restraints observed in the ^1^H-^1^H NOESY experiments (Figure modified from CcpNmr Analysis output). (**B**) Secondary chemical shifts (Δδ) of H_N_, H_α_, C_α_, and C_β_ relative to random coil chemical shifts in DMSO^20^. (**C**) Secondary structure prediction by CSI (calculated from Δδ of H_α_, C_α_, and C_β_; cutoffs for helical character^20^ illustrated by dashed red lines in (**B**) and DANGLE (CcpNmr Analysis).

Through consideration of NOE contacts characteristic of helical structuring^16^ alongside the observed Δδ values of H_α_, C_α_, and C_β_ nuclei^17–19^ calculated relative to random coil chemical shifts in a low dielectric environment^20^, a helical segment is clear over residues ^18^Ala-Lys^21^ (Fig. 2, corresponding to amino acids 375–386 of NHE1). In the N-terminal portion of the TM XI peptide, ^1^Lys to ^8^Val (containing amino acids 360–365) showed some degree of helicity according to Δδ-based predictions (Fig. 2), but this was not continuous. Similarly, DANGLE, which predicts secondary structuring based upon comparison of Δδ relative to random coil values in 8 M urea^22^ to database inferences for a given sequence and structuring, predicted a long helical stretch in the C-terminal half of the TM IX segment and a short helical stretch in the N-terminal portion (Fig. 2). Unambiguous canonical helical NOE contacts were not observed in the N-terminal region, as chemical shift overlap was more problematic within this portion of the peptide.

### Mutagenesis

**Figure 3:**
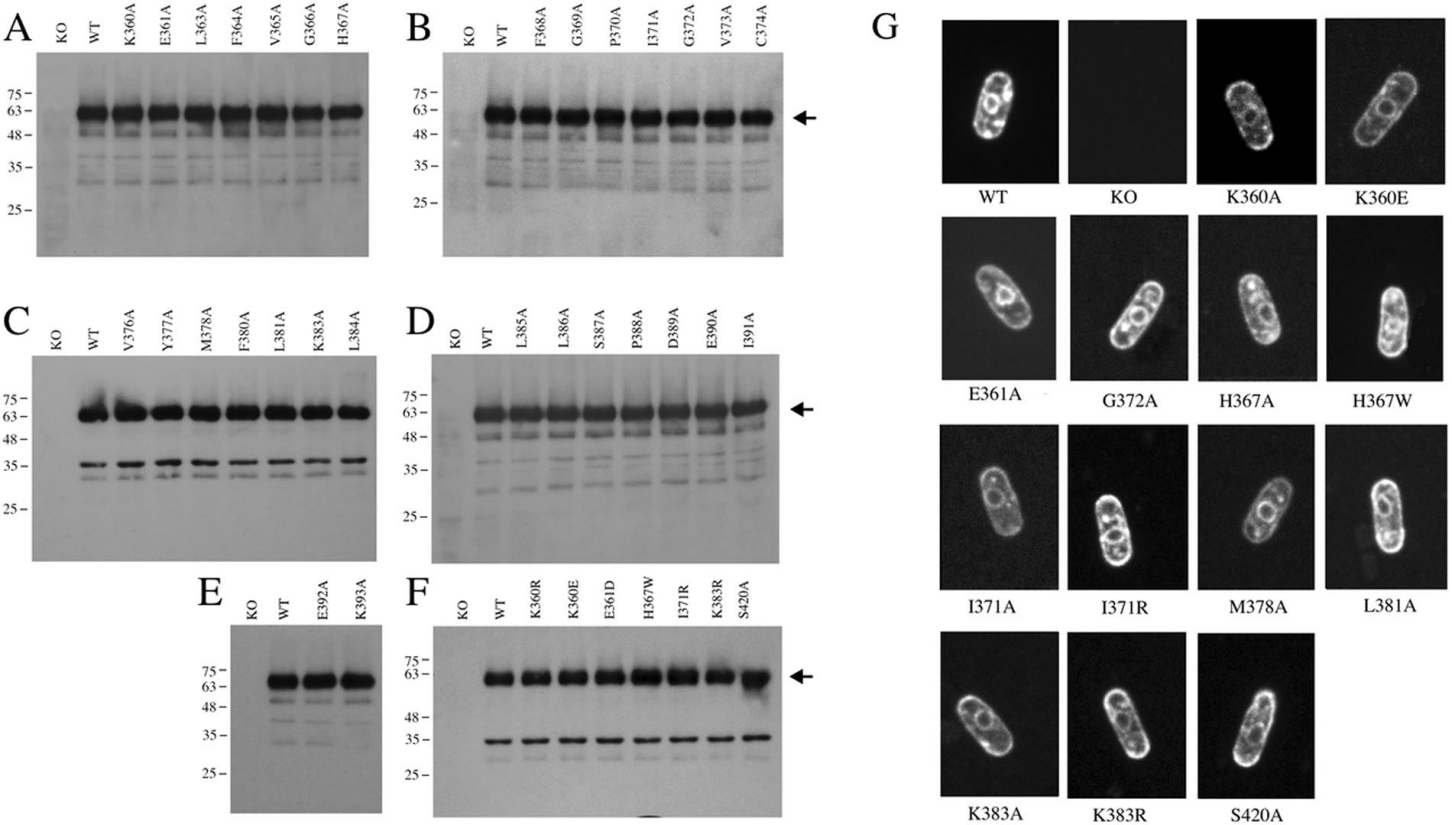
(**A**–**G**) Expression and localization of various *Sp*NHE1 proteins. (**A–F**) Western blot analysis of expression of *Sp*NHE1 proteins was from cell extracts from *S. pombe* strains expressing either wild type *Sp*NHE1 or various *Sp*NHE1 mutants. It was blotted with anti-GFP antibody as described in the materials and methods. Arrow indicates approximate location of full length *Sp*NHE1 with the GFP tag. (**A–E**) first round of mutagenesis to alanine. (**F**) Second round of mutagenesis to amino acids other than Ala. (**G**) Confocal microscopy of wild type *Sp*NHE1 and selected *Sp*NHE1 mutants in *S. pombe*. Exponentially grown cells were harvested and used directly for live cell imaging of GFP fluorescence.

We made two sets of mutants in the putative TM XI to analyze the importance of amino acids in this region. The first set was an alanine scan of residues 360 to 393. The second set involved particular mutations of specific residues of this region, plus amino acid Ser^420^, which we hypothesized was associated with TM XI (Supplementary Table 1, Table 1). Initial experiments used western blot analysis to determine if the wild type and mutant *Sp*NHE1 proteins were produced. Figure 3A–F demonstrates the results. All of the mutant proteins were expressed in *S. pombe* in amounts similar to that of the wild type. There was some small varying amounts of degradation of the *Sp*NHE1 protein; however, the principal form of the protein was approximately 65 kDa in size. It was expressed at similar levels to the wild type *Sp*NHE1 in all the mutants. The knockout strain that was not transfected with *Sp*NHE1 showed no immunoreactivity. We also examined the localization of the wild type and mutant *Sp*NHE1 proteins with defects in activity (see below). The wild type protein shared the same localization (Fig. 3G) we have reported^23,24^. It was present on the plasma membrane and also at an intracellular location that may be perinuclear. The mutant *Sp*NHE1 proteins had the same apparent localization as the wild type protein.

**Table 1:** Summary of growth of yeast strains containing wild type, or mutant *Sp*NHE1 in liquid (L) or solid (S) media containing NaCl or LiCl. Mutations to amino acids are indicated. The measured or modeled propensity of an amino acid to be in a helix or extended conformation is indicated.

Type	NaCl (L)	LiCl (L)	NaCl (S)	LiCl (S)	Group	NMR	model
WT	**+++**	**+++**	**+++**	**+++**		—	—
KO	—	—	—	—		—	—
K360A	—	**+++**	**+**	**++**	*	**h**	**H**
E361A	**+**	—	**+**	**+**	***	**h**	**H**
L363A	**+++**	**+++**	**+++**	**+++**		**h**	**H**
F364A	**++**	**++**	++	+	#	**h**	**H**
V365A	**+++**	+++	++	+++		**h**	**H**
G366A	**+++**	+++	++	++		**E**	**H**
H367A	**+**	+	+	+	***	**E**	**H**
F368A	**+++**	**+**	**++**	**+**	#	**E**	**H**
G369A	**+++**	**++**	**+++**	**++**		**E**	**H**
P370A	**+++**	**++**	**+++**	**++**		**E**	**E**
I371A	**++**	—	**++**	—	**	**E**	**E**
G372A	**++**	—	**++**	—	**	**E**	**E**
V373A	**++**	**+++**	**+++**	**++**		**E**	**E**
C374A	**+++**	**+++**	**+++**	**++**		**E**	**H**
V376A	**+++**	**+++**	**+++**	**++**		**H**	**H**
Y377A	**++**	**++**	**++**	**+**	#	**H**	**H**
M378A	**+++**	—	**++**	**+**	**	**H**	**H**
F380A	**++**	**++**	**++**	**+**	#	**H**	**H**
L381A	**+++**	—	**+++**	**+**	**	**H**	**H**
K383A	**+**	**+**	—	**+**	***	**H**	**H**
L384A	**+++**	—	**++**	**++**	#	**H**	**H**
L385A	**+++**	**+++**	**+++**	**++**		**H**	**H**
L386A	**++**	**++**	**+**	**++**	#	**H**	**E**
S387A	**+++**	**++**	**+++**	**+**		—	**E**
P388A	**++**	**++**	+++	+++		—	**E**
D389A	**++**	+++	+++	++		—	**E**
E390A	**+++**	+++	+++	++		—	**E**
I391A	**+++**	+++	++	++		—	**E**
E392A	**+++**	+++	+++	++		—	**E**
K393A	**++**	+++	+++	++	#	—	**E**
K360R	**+++**	+++	++	+++			
K360E	**++**	+++	+	++	*		
E361D	**+++**	+++	++	+++			
H367W	**++**	+++	++	++	#		
I371R	—	—	—	—	***		
K383R	**+**	+	+	++	***		
S420A	—	+	—	+	***		

^*^Cells with increased relative sensitivity to NaCl than to LiCl; **cells with increased relative sensitivity to LiCl than to NaCl; ***cells with sensitivity to both LiCl and NaCl; #cells with minor changes in sensitivity to LiCl and/or NaCl; h, NMR results suggest possible helicity; H, in a helical conformation; E, predicted or shown to be in an extended conformation. −, No growth; **+**,**++**,**+++**, increasing amount of growth with **+++**, indicating growth equivalent to wild type. −, not applicable or not measured.

We (Supplementary Figure 3) then examined the ability of the wild type or mutant *Sp*NHE1 protein to restore growth in salt containing medium. Supplementary Figure 3A–D illustrate the growth curves in NaCl containing medium. Wild type *Sp*NHE1 protein restored growth to the knockout strain. Cells were able to grow robustly in solution containing 500 mM NaCl. In contrast, the knockout strain showed no growth in 500 mM NaCl and compromised growth in 200 mM NaCl. Mutation of several amino acids caused impairment of growth in high concentrations of NaCl. This was most notable with the K360A mutation (Supplementary 3A), which was almost comparable to the knockout strain. Mutations H367A and K383A also caused growth to be largely compromised in 500 mM NaCl. Several other mutants had more minor effects on the ability to tolerate higher concentrations of NaCl including E361A (summarized in Table 1). In the second round of mutagenesis, with mutations to other amino acids, the proteins with mutations K360R, E361D and H367W conveyed NaCl resistance at levels equivalent or similar to that of the wild type protein. There was also a minor effect with K360E. The mutant proteins I371R, K383R and S420A did not convey sodium tolerance (Supplementary Figure 3E).

**Figure 4:**
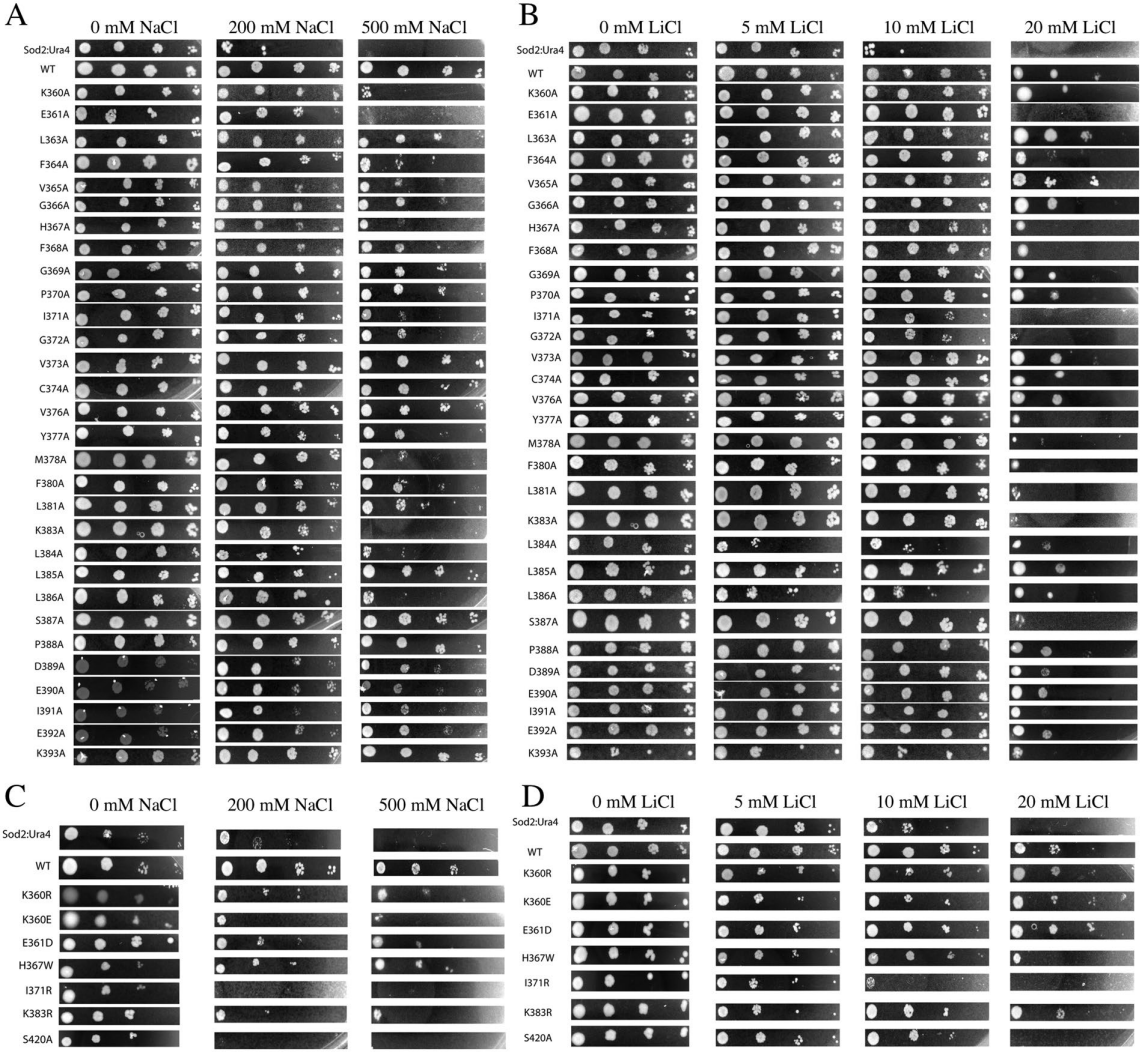
Growth of wild type and mutant *Sp*NHE1 containing *S. pombe* transformants on solid media. Samples of the various strains were from stationary phase cultures and were serially diluted 10-fold repeatedly. They were then spotted onto minimal media plates supplemented with NaCl (**A**,**B**) and LiCl (**C**,**D**) at the indicated concentrations. Plates were incubated at 30 °C for 3 days. (**A**) Growth of controls and alanine scanning mutants on NaCl containing media. (**B**) Growth of controls and other mutants on NaCl containing media. Panels illustrating growth on plates were supplemented with NaCl at the concentrations indicated. (**C**) Growth of controls and alanine scanning mutants on LiCl containing media. (**D**) Growth of controls and other mutants on LiCl containing media. Panels illustrating growth on plates were supplemented with LiCl at the concentrations indicated. Sod2::ura4 refers to *S. pombe* with the *Sp*NHE1 knockout described earlier^9^. WT refers to the sod2::ura4 with wild type *Sp*NHE1 expressed from pREP-41sod2GFP as described earlier^23^. Other designations refer to *Sp*NHE1 expressed from the plasmid pREP-41sod2GFP with the indicated mutation. Results are typical of at least 3 experiments.

We also examined the same set of mutants for their ability to grow on solid media supplemented with NaCl. Figure 4A,B illustrates the results of growth on solid media with NaCl for the wild type and mutants, and the results are summarized in Table 1. The pattern of effects of the mutations on the ability to confer salt tolerance was largely the same as in liquid media. Mutant K360A and K383A proteins were unable to convey NaCl tolerance. Additionally, the H367A and L386A mutant proteins were largely ineffective in conferring NaCl tolerance. More minor effects were shown by the F364A, I371A, G372A, Y377A, M378A, F380A, L384A, and I391A (Table 1). Proteins with the mutation K360E, I371R, K383R and S420A were also defective in conveying salt tolerance on solid media, similar to the effect in liquid media.

The sensitivity of the mutants to LiCl was also examined in liquid and solid media (Supplementary Figure 4, Fig. 4C,D). Many mutants, with alanine mutations that were sensitive to NaCl, were also sensitive in similar degrees to LiCl. This was the case for F364A, H367A, Y377A, K383A and L386A. However, some mutants showed acute differences in their ion sensitivity. This was most notable for the K360A mutant strain, which lost virtually all NaCl tolerance, but was able to grow relatively well in the presence of external LiCl. The adjacent amino acid E361A had sensitivity to both LiCl and to NaCl. The cells with the mutant protein containing the amino acids I371A, G372A, M378A, and L381A were all more sensitive to LiCl than NaCl. Of the second group of mutations, proteins with the I371R, K383R and S420A did not confer resistance to LiCl, similar to what occurred with NaCl. The K360E mutant protein, conferred reasonable resistance to LiCl, in contrast to its weaker ability to do the same for NaCl (Summarized in Table 1).

## Discussion

Na^+^/H^+^ exchangers were earlier reported to have a unique fold that is hypothesized to be critical in transport by these proteins and is believed to part of the ion translocation core. The key features that have been noted are in the critical TM segments IV and XI. Both segments have been shown to be discontinuous helices that cross over each other in anti-parallel fashion with their crossing point within the lipid bilayer. Other amino acids neutralize the dipoles of the helices within the bilayer^11,14^. This arrangement was initially reported in NhaA of *E. coli* ^11^, and has subsequently been shown in *Thermos thermophilus* (NapA)^12^, in the archaeal Na^+^/H^+^ exchanger of *Methanococcus* MjNhaP1^25^ and in *Pyrococcus abyssi* (PaNhaP)^13^. In PaNhaP and NapA, this structure is formed by helices 5 and 12 of a 13 TM segment protein, in contrast to the 12 TM segment protein NhaA.

While significant progress has been made in these species, in others there is not as much known about other NHE’s. Notably, whether the same mechanisms of transport and protein conformation are maintained across species is not known. The *S. pombe* protein *Sp*NHE1 is thought to be electroneutral in contrast to NhaA, which would certainly require some structural and functional differences^8,11^. From the sequence alignment, we noted that this yeast plasma membrane NHE also has some unique substitutions in TM XI. For example, Pro^370^ is exclusively present in TMXI of *Sp*NHE1 and yeast. Additionally, a conserved histidine (His^367^) in TMXI segment is unique to the yeast plasma membrane NHEs. Also, a hydrophobic amino acid (Ile^371^) is present in yeast plasma membrane NHE1, which is Arg in other NHE1’s. *Sp*NHE1 may therefore have a novel or altered mechanism of Na^+^ transport. *Sp*NHE1 works to bring Na^+^ out of the cell^9^ in contrast to mammalian NHE1. These conserved unique substitutions may play a key role in function.

We therefore examined the role of putative transmembrane segment XI in *Sp*NHE1 which we hypothesized was critical in function. In this species, this protein is the key sodium tolerance protein. Its deletion causes salt sensitivity that makes it ideal to assay *Sp*NHE1 function in live cells. We have earlier studied amino acids of this protein and defined several residues critical or important for function^9,15^. The 27 amino acids ^360^KEALFVGHFGPIGVCAVYMAFLAKLLL^386^ comprise a hydrophobic segment that was chosen for study. There were varied degrees of similarity of this segment to Na^+^/H^+^ exchangers of other species (Fig. 1A), with other yeast species showing the greatest degree of similarity. In *Thermos thermophilus* (NapA), this segment of *Sp*NHE1 aligned with TM 12, which is thought to be part of the ion translocation core. This was also the case with TM XI of *E. coli* NhaA, though in neither case was the similarity very high. In the case of MjNhaP1 and PaNhaP, the alignment with the corresponding regions of the ion translocation core is stronger.

In an earlier study we generated a *Sp*NHE1 homology model (Fig. 1B,C) based on the *E*. *coli* NhaA structure and a comparative sequence alignment^15^. This model was validated using mutagenesis and structural analysis of TMIV^15^. Based on that model, a helical prediction of TMXI segment was made for residues 360–368 and residues 374–385, with an intervening non-helical segment (Fig. 1B, Table 1). To examine the structure of this transmembrane segment directly, we characterized a 27 amino acid peptide corresponding to residues 360–386 of *Sp*NHE1 using solution-state NMR spectroscopy. It has been previously demonstrated that isolated peptides of TM segments contain most of the required structural information needed to form their native structures in membranes. For example, the solution structure of isolated segments of bacteriorhodopsin corresponded very well to the crystal structure of the protein^26–28^. Our results suggest that amino acids Lys^360^ to Val^365^ exhibited chemical shifts predominantly consistent with helicity, while a continuous helical segment was strongly indicated over residues Ala^375^-Ile^391^. These results are summarized in Table 1. This was essentially in agreement with the earlier modeling of the protein^15^, and is consistent with a break in helical character around the central proline residue of the segment^29,30^. A proline in the midst of a transmembrane segment of Na^+^/H^+^ exchangers has earlier been suggested to cause an extended region within the lipid bilayer^31^. Overall, these results suggest that this TM segment retains the basic structure of the ion translocation domain. That is, a helix-extended region-helix motif. While the helical propensity of the N-terminal region of the segment was less pronounced, it was nonetheless present. It may be that interactions with hydrophobic regions of other transmembrane segments or lipids could stabilize a slightly more helical conformation^32^.

We next asked the question, what is the functional importance of the individual amino acids of this transmembrane segment? Alanine scanning mutagenesis was used. Alanine has a small side chain that can substitute for most amino acids without disrupting the protein and at the same time alter the side chain. This kind of scan has been used earlier on *Sp*NHE1^15^ and other proteins^21,33^. All of the amino acids of this TM segment were mutated to alanine, aside from those that were already present as alanine. We then examined the ability of the expressed protein to rescue salt tolerance in the yeast knockout strain. Mutation to alanine (or other amino acids) did not affect the level of *Sp*NHE1 protein expression. This result is consistent with what we observed earlier when amino acids of TM IV were mutated^15^. In contrast, when the mammalian NHE1 protein is mutated, many mutations affect both expression levels and targeting^34^. It appears as though that, at least for this class of proteins, in yeast, expression and targeting of the protein is more robust than for the mammalian NHE1 protein.

**Figure 5:**
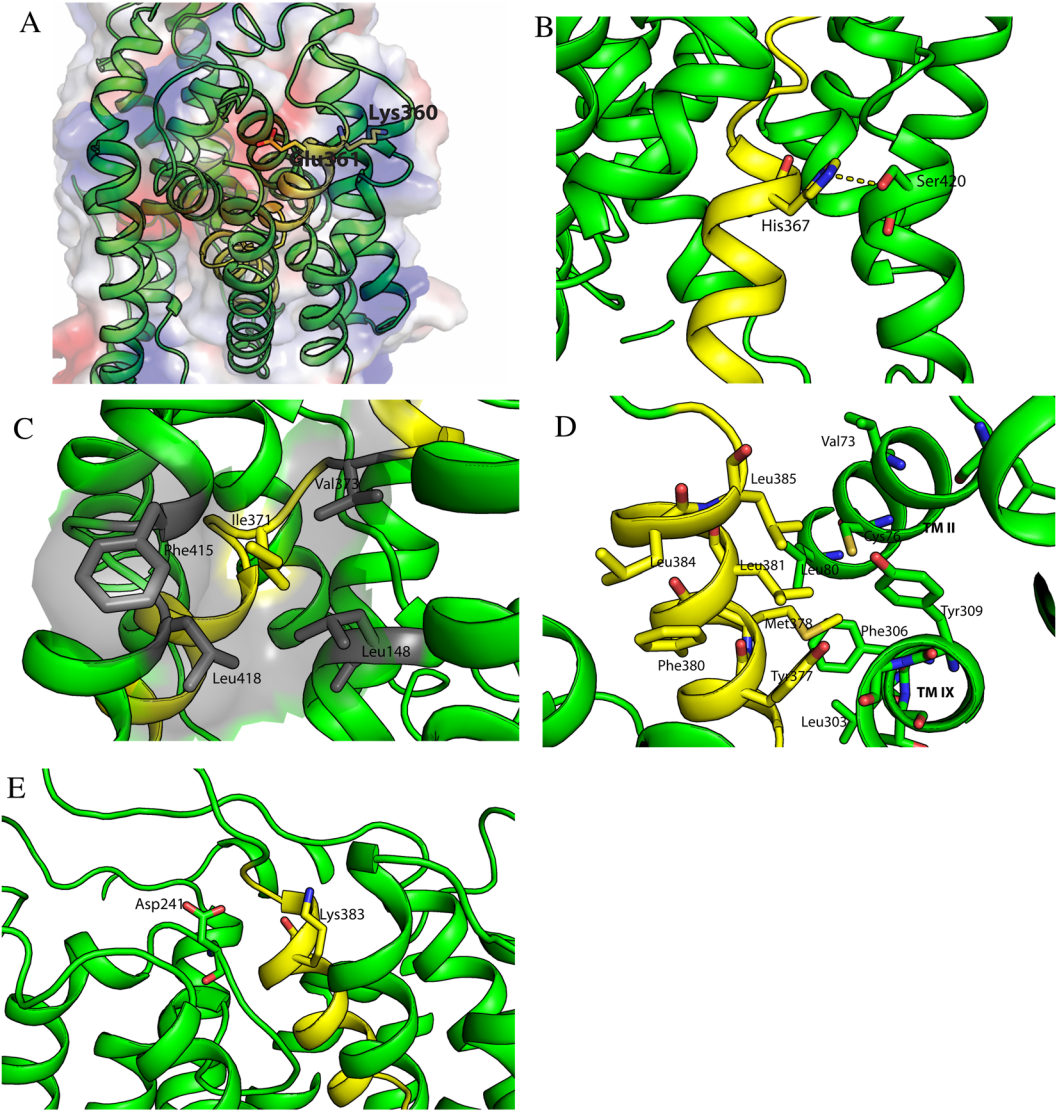
Molecular models of selected regions of *Sp*NHE1 based on^15^. TM XI shown in yellow. Other segments in green. (**A**) Illustration of position of Lys^360^ and Glu^361^ on the external face of *Sp*NHE1. (**B**) Position of Ser^420^ relative to His^367^. (**C**) Position of Ile^371^ relative to residues Leu^148^, Phe^415^, and Leu^418^. (**D**) Position of M378A, L381A, L384A and L386A relative to other hydrophobic resides. (**E**) Putative association of Asp^241^ and Lys^383^.

We consider the effects of the amino acid substitutions in the light of the *Sp*NHE1 model (Fig. 1B,C) and some of the amino acid interactions predicted in this model. It should be noted, that the model has yet to be definitely proven, nevertheless, some insights may be gained by this analysis. Nine of the mutations to alanine had more major effects on conferring resistance to Li, Na or both cations. Beginning from the N-terminus, there were two amino acids, Lys^360^ and Glu^361^, that, when mutated, affected Na and Li tolerance. According to the previously hypothesized model, these two amino acids could be on or near the intracellular face of the membrane^15^. Mutation of these residues affecting ion specificity could be because they are involved in coordination of the internal cation, especially for negatively charged glutamic acid. Figure 5A illustrates their position on the surface of the protein. Replacement of the acidic Glu^361^ with aspartic acid largely restored activity, supporting this hypothesis. It is also notable that this amino acid is largely conserved across many species (Fig. 1), though in some species it is replaced by an aspartate residue. Notably though, *T. thermophilus* NapA and *E. coli* NhaA do not contain an acidic residue at this location. A predicted topological assignment of this residue is at the cytoplasmic side and is located in close proximity to Glu^165^ and Asp^355^. Asp^355^ is conserved among the yeast species while Glu^165^ is not conserved at all. Therefore, it is more likely that Glu^361^ is directly involved in proton transport together with Asp^355^ among the yeast group.

The side chain of the positively charged Lys^360^ is predicted to point away from the central cavity (Fig. 5A)^15^. Its mutation affected ion specificity and this may be through an affect on the coordination sphere indirectly through alterations with other amino acids, that affect the sphere structure. Replacement of Lys^360^ with a positively charged arginine largely restored function. Replacement of Lys^360^ with a negatively charged glutamic acid had an intermediate effect, demonstrating that the charge requirement is not absolute (Table 1). This amino acid is largely conserved across species (Fig. 1), though often replaced with arginine. A basic amino acid is present in NhaP1, NhaP, and NapA, though NhaA of *E. coli* is different, which may reflect specific differences in function and activity of this protein.

The next critical residue in *Sp*NHE1 function was amino acid His^367^. Mutation of this residue to Ala severely impaired the ability of the protein to rescue salt tolerance, confirming an earlier result^9^. We have previously also shown that the H367R mutant is unable to transport Na, whereas the H367D mutant was also defective shifting the pH optimum to a more alkaline range. Analyzing the predicted *Sp*NHE1 structure^15^ suggests that the imidazole ring can make a hydrogen bonding interaction with hydroxyl group of Ser^420^ (or Thr), which is present in TM XII of *Sp*NHE1 (Fig. 5B). We therefore mutated Ser^420^ to an alanine residue and examined the effect on protein function. The results demonstrated that this mutant protein is defective (Table 1). This supports the suggestion that there is an association that affects function. We also noted that in plant SOS1 protein, the equivalent position of His^367^ is replaced with a tryptophan. Therefore, we examined if the H367W mutant can retain the protein’s activity. We found that the H367W containing cells are not sensitive to Na and Li, supporting the idea that that the charge distribution on the imidazole nitrogen of histidine is responsible for the activity. Tryptophan has a nitrogen-containing ring in its indole side chain, which may perform a similar role to the imidazole side chain of histidine. Interestingly this residue is located in the discontinuous helix region.

More towards the center of the TM segment, both the I371A and G372A mutants had similar effects on salt tolerance, causing defects in both Na and Li tolerance, with a more pronounced effect on Li tolerance. Both of these residues are within the extended region of the TM segment (Fig. 2). Ile^371^ is present in yeast plasma membrane NHEs and *E*.*coli* NhaA and is within the sequence GPIG. Sequence alignment (Fig. 1) identified the corresponding sequence motif in human NHE1, with an Ile to Arg substitution as ^455^GGLRG^459^. The GLRG sequence is also observed in some plant antiporters. At the same time, in some other antiporters (MjNhaP1, PaNhaP, and TthNapA) the corresponding sequence was GPRG. The NMR structure of a peptide of this region of human NHE1 suggested that this part of the protein is in an extended conformation and is not helical^35^. Here, in the absence of a proline comparable to Pro^370^ of *Sp*NHE1, the two glycines (Gly^445^ and Gly^446^) may maintain flexibility.

Examination of the *Sp*NHE1 model^15^ suggests that Ile^371^ may have hydrophobic interactions with some surrounding residues like Leu^148^, Phe^415^, and Leu^418^ (Fig. 5C). The corresponding isoleucine in *Ec*NhaA is Ile337 and is engaged in hydrophobic interactions^11^. Mutation of Ile^371^ to Arg severely compromised activity of the protein (Table 1), supporting the contention that hydrophobic interactions are important in this location.

The G372A mutation had an effect similar to that of I371A, causing defective Na and Li tolerance. The exact cause of the effect is uncertain though the change in specificity does suggest an effect on ion coordination. A change from Gly to Ala might increase helical character, which could affect the extended conformation of this region. Glycine tends to allow flexibility, possibly acting as a hinge point-flexibility, and is rarely in a helix^36–38^. It may be that mutation to Ala interferes with the flexibility that Gly may induce in this region and that this affects ion coordination.

A segment of amino acids from Val^377^ to Leu^384^, when mutated, all had either minor or major effects on the ability of *Sp*NHE1 to confer salt tolerance. This is thought to be either a partially extended and helical region or a helical region (Table 1). The effect of mutations Y377A, F380A, L384A (and nearby L386A) were minor. The mutants M378A, L381A, L384A and L386A all yielded varying degrees of a defective *Sp*NHE1 protein. Modeling^15^ of *Sp*NHE1, suggests that these residues are in close proximity to a cluster of conserved hydrophobic amino acids. This includes the C-terminal portion of TM II (residues include Ile^78^, Val^79^, Leu^80^, and Val^82^) and the C-terminal portion of TM IX (residues include Phe^305^, Phe^306^, Tyr^309^) (Fig. 5D). The effect of these mutations may be due to reduced hydrophobic interactions between TM XI and these hydrophobic amino acids. It is interesting to note that in NhaA the equivalent helices are involved in helix-helix interactions as part of the scaffolding dimerization domain of NhaA. The recent structure of *Tt*NapA also suggests that the C-terminal halves of TMIV and TMXI mediate scaffolding interactions with the dimerization domain^39^. The mutants M378A, L381A, L384A and L386 are all localized to the C-terminal half of TM XI of *Sp*NHE1. If this region of the TM segment serves a similar role in *Sp*NHE1 to that of the C-terminal of TM XI of *Tt*NapA, this may explain their importance in function. They could be affecting dimerization of *Sp*NHE1 through hydrophobic interactions with other amino acids. We have not demonstrated dimerization of *Sp*NHE1, though this has been shown in the related protein *At*SOS1^40^. SOS1 dimerization facilitates Na^+^ transport^40^. Similar hydrophobic interactions through the C-terminal half of TM-XI of *Sp*NHE1 could affect Na^+^ transport through effects on dimerization.

The K383A mutation had a dramatic effect on activity of the protein and the K383R substitution only partially recovered activity. The larger size of this side chain may have affected interaction with other amino acids. This amino acid is conserved across the yeast and plant antiporters but not in some other species. Examination of the position of Lys^383^ in the model of *Sp*NHE1^15^ suggests that this residue may interact with Asp^241^ (Fig. 5E). We have previously shown that mutation of Asp^241^ to Asn interferes with *Sp*NHE1 function^9^. This supports the suggestion that there is an interaction between these amino acids.

Amino acid residues 387 to 393 showed no large effects with mutation to alanine. We earlier^10^ showed that the mutations E390Q and D389N did not affect salt tolerance. This region seems relatively insensitive to mutation.

Overall, our results in this study can be compared with the human NHE1 protein. In *Sp*NHE1, we found that the non-helical discontinuous part of TM XI spanned about 10 amino acid residues. A similar study of a peptide of TM XI of human NHE1^35^ suggested that a comparable discontinuous region of TM IX was about 5 amino acids. Landau *et al*.^41^ suggested a model of human NHE1 based on the crystal structure of *E. coli* NhaA. In this model amino acids 447–470 are proposed to be human TM XI and an even smaller discontinuous region is suggested in mid membrane centered around amino acid Leu^457^.

Another difference between human NHE1 and *Sp*NHEI is that, *Sp*NHE1 TM-XI does not contain an Arg (or Lys) at the center of the segment whereas human NHE1-TMXI contains Arg^458^ there. The side chain of such a residue could be attracted to phospholipid head groups and could aid in conformational switching^42^. In the absence of an arginine, *Sp*Nhe1 TM-XI contains His^367^, which is located at the N terminus of the discontinuous region, and can attain a positive charge over certain pH ranges. The H367R mutant is incapable of transport confirming this is an important residue. However, as noted above, this residue might also interact with other amino acids such as Ser^420^. Three important residues L457, I461, and L465 in human NHE1-TMXI, have also been identified based on cysteine mutagenesis and are located either at the discontinuous region or at the C-terminal half of the TM^35^. An analogous effect of mutagenesis was observed for the C-terminal half of *Sp*Nhe1 where the mutations of the residues to alanine are moderately or highly affected protein activity.

In summary, based on NMR analysis, the deduced structure of a peptide of TMXI of *Sp*NHE1 was that of a putative helix-extended region-helix. The functional data suggested that there were several more important regions on the transmembrane segment, where alteration to Ala compromised the protein’s activity. When correlating effects of the mutations with molecular modeling of the protein, we came to the following putative roles of these amino acids on the protein. Glu^361^ may be involved in cation coordination on the cytoplasmic face of the protein. His^367^, may be in association with Ser^420^ and this is an important functional association. Ile^371^ and Gly^372^ are important in function. Ile^371^ may have important hydrophobic interactions with other residues on transmembrane segments and Gly^372^ may be important in maintaining an extended conformation of this region. A unique feature of *Sp*NHE1 that we demonstrated, was that His^367^ and Ile^371^ are important functional residues of *Sp*Nhe1 and are conserved across related species. Residues from Val^377^ to Leu^384^ are important in function and may be important in hydrophobic interactions with other amino acids. Lys^383^ of this region is also important in function and may be important in other ionic-electrostatic interactions with other amino acids. We suggest that TM XI of *Sp*NHE1 has a similar structure and function to TM XI of *E. coli*, forming part of the ion translocation pathway that is characteristic of Na^+^/H^+^ exchangers. It is possible that some mutations affect *Sp*NHE1 indirectly, disrupting protein structure. Interpretations such as Glu^361^ acting through an effect on cation coordination are based on molecular modeling, which remains to be confirmed by determination of the three dimensional structure of the protein.

## Methods

### Materials

Restriction enzymes were obtained from New England Biolabs, Inc. or In vitrogen. PWO DNA polymerase was from Roche Applied Science (Roche Molecular Biochemicals, Mannheim, Germany). Synthetic DNA for mutagenesis was from Integrated DNA Technologies. A synthetic peptide corresponding to the putative TMIX (sequence Acetyl-KKKEALFVGHFGPIGVCAVYMAFLAKLLLKKK-amide) was purchased from the Alberta Proteomics and Mass Spectrometry Facility and was purified by HPLC and the identity verified by Mass Spectrometry. Deuterium oxide (D_2_O; 99.9% D); D_2_O with 1% sodium 2,2-dimethyl-2-silapentane-5-sulphonate (DSS); and, deuterated dithiothreitol (DTT-d_6_; 98% D) and DPC-d_38_ (98% D) were purchased from C/D/N Isotopes (Pointe-Claire, Quebec, Canada). CD_3_COOH (>99% D) was purchased from Sigma Aldrich (Oakville, Ontario, Canada).

### Strains and media

*S. pombe* with the *NHE1* gene disrupted (sod2::ura4) was used as a host to reintroduce wild type and mutant *Sp*NHE1 protein^9^. The sod2::ura4 strain was maintained on low sodium minimal KMA medium or yeast extract adenine (YEA) as we described earlier^8,9^. KMA medium contains potassium hydrogen phthalate, 3 g; K_2_HPO_4_, 3 g; yeast nitrogen base without amino acids, 7 g; glucose, 20 g; and adenine, 200 mg (per 1 liter). Leucine at 200 mg/l was added to maintain the *sod2::ura4 leu1-32* strain wherever indicated and all media was buffered using 50 mM MES/Citrate and pH adjusted to 5.0 with sodium free KOH. Wherever indicted NaCl or LiCl was added to the media at the indicated concentrations. The plasmid pREP-41sod2GFP was used to express the *Sp*NHE1 protein as described earlier^23^. pREP-41sod2GFP contains the full length *Sp*NHE1 gene with a C-terminal GFP tag separated by a nine amino acid Gly-Ala spacer. The GFP protein contains the Ser65Thr mutation and an NdeI site was removed by silent mutation to assist in cloning.

Transformation of the plasmid (and mutant forms) was into the sod2::ura 4 strain by electroporation^43^. Briefly, cells were grown in 100 ml of KMA (with Leucine) media at 30 °C with vigorous shaking until the OD600 reaches to 0.5 to 1.2. Cells were then incubated in ice for 15 min and harvested by centrifuging at 3500 × g for 5 min at 4 °C. The supernatant was discarded and the pellet was resuspended in 200 ml of ice cold water. Washed cells were collected by centrifugation as above and further washed with 50 ml of ice-cold 1.0 M sorbitol twice. Finally, the cell pellet was resuspended in 1 ml of 1.0 M sorbitol. Resuspended cells were divided into 200 μL of aliquots and mixed with 0.1 ng of purified DNA. The cell-DNA mixture was transferred to a 0.2 cm electroporation cuvette pre-incubated in ice. After 5 min incubation an electric pulse was applied according to the Gene Pulser II (Bio-Rad) specifications. Immediately after pulsing, cells were resuspended in 800 μL of 1.0 M sorbitol and incubated at 30 °C without shaking for 1 hr. Cells were then centrifuged and spread in KMA agar containing 1.0 M sorbitol without leucine for growth and selection. Colonies appeared after 3–4 days.

Growth of transformed strains was in liquid and solid media. For growth curves in liquid media 5 × 10^6^ cells were taken from an overnight exponentially growing culture. This was inoculated into 2.5 ml of fresh liquid media. *S. pombe* containing the pREP-41sod2GFP plasmid and mutants were routinely grown in medium in the absence of thiamine. Cultures were grown at 30 °C in a rotary shaker with constant agitation. The A_600_ was determined at the indicated times. Growth curves were determined a minimum of three times and results are the mean + /− SE.

Growth on plates was examined in agar with KMA medium containing leucine supplemented with either NaCl or LiCl at the indicated concentrations. The pREP-41sod2GFP plasmid without mutations^23^ was used as a control.

### Site-Directed Mutagenesis

Mutations to *Sp*NHE1 were made by PCR amplification of the pREP-41sod2GFP plasmid. The mutations were designed to create or remove a restriction enzyme site as described earlier^31^. DNA sequencing was used to confirm the accuracy of the mutations and the fidelity of DNA amplification. Supplementary Table 1 summarizes the mutations made to *Sp*NHE1.

### Western Blotting of *Sp*NHE1

Western blot analysis was used to compare levels of *Sp*NHE1 expression in wild type and mutant *Sp*NHE1 protein^31^. Cell lysates were made from 50 ml of cultures of yeast transformants. Yeast cells were grown at 30 °C in KMA medium to an OD600 of 2. Cells were pelleted (3500 × g, 10 min) and were then washed with double distilled water and resuspended in a lysis buffer consisting of 50 mM Tris-HCl, pH 8.0, 5 mM EDTA, 1 mM dithiothreitol and a protease inhibitor cocktail^44^. Cells were then lysed using a Bullet Blender^®^ using 0.5 mm zirconium oxide beads, speed of 10 × 40 minutes. In some cases they were lysed by passage through an emulsiflex homogenizer at a pressure of 25000 psi. Unbroken cells were pelleted by centrifugation at 3500 × g for 5 min, and the supernatant was centrifuged (14000 × g × 10 min). Enriched membranes of the supernatant were then pelleted at 100000 x g for 1 h, and were resuspended in a small volume of 50 mM Tris-Cl pH 8.0, 150 mM NaCl, 5 mM EDTA, 1 mM EGTA, 1.0% (v/v) NP-40, 0.5% (w/v) deoxycholate and 0.1% (w/v) SDS. Equal amounts of up to 25 μg of each sample were resolved on SDS/polyacrylamide gels (10%). Western blotting of nitrocellulose transfers used a primary antibody of anti-GFP polyclonal antibody (a generous gift of Dr. Luc Berthiaume, Dept. of Cell Biology, University of Alberta). The secondary antibody was peroxidase-conjugated goat anti-mouse antibody (Bio-Can, Mississauga, Canada). Protein reactivity was detected using X-ray film via the Amersham enhanced chemiluminescence western blotting and detection system.

### *Sp*NHE1 sequence Alignment

*Sp*NHE1 multiple sequence alignment was performed using MAFFT^45^. Representatives of different groups included, sequences of yeast, fungi, plant, mammalian, and bacterial plasma membrane Na^+^/H^+^ exchangers were included. From yeast and fungi; *S*. *pombe* NHE1 (**NP_592782.1**), *S*. *pombe* Sod22 (**NP_594194.1**), *Candida albicans* CNH1 (**XP_710352.1**), *C*. *albicans* Cnh1p (**AAL24468.1**), *Debaryomyces hansenii* Nha1p (**CAI45290.1**), *Saccharomyces cerevisiae* Nha1p (**NP_013239.1**), *Zygosaccharomyces rouxii* Sod22 (**XP_002497045.1**), *Z*. *rouxii* Nha1 (**XP_002497801.1**), *Yarrowia lipolytica* Nha1p (**XP_501299.1**), *Y*. *lipolytica* Nha2p (**XP_503447.1**) are included. *Methanocaldococcus jannaschii* NhaP1 (**NP_247021.1**), *Pyrococcus Abyssii* NhaP (**CAB50204**), *Thermus thermophiles* NapA (**YP_144738**), and *Escherichia coli* NhaA (**WP_000681354**) are included as acheal and bacterial members. Plant *Arabidopsis thaliana* SOS1 (**AAL32824**), *Brassica napus* SOS1 (**AGA37213.1**), and *Theobroma cacao* SOS1 (**XP_007045406.1**) are included. Finally mammalian *Homo sapiens* NHE1 (**NP_003038.2**), *Pan troglodytes* NHE1 (**XP_016812591**), and *Rattus norvegicus* NHE1 (**AAA98479**) are also included. The alignment was prepared using ESpript^46^.

### Nuclear magnetic resonance spectroscopy

The NMR sample was prepared by dissolving 1 mM synthetic peptide in a 95% H_2_O 5% D_2_O solution containing 20 mM CD_3_COO^−^, 1 mM DSS, 1 mM NaN_3_, 10 mM DTT-d_6_, and 150 mM DPC-d_38_. pH was then adjusted to 5.00 ± 0.05. All NMR experiments (natural abundance ^1^H-^13^C HSQC, ^1^H-^1^H TOCSY and ^1^H-^1^H NOESY) were acquired at 30 °C using an Avance III 700 MHz spectrometer equipped with a 5 mm triple resonance inverse cryoprobe with a z-axis gradient (Bruker Canada) at the Biomolecular Magnetic Resonance Facility, National Research Council, Halifax, NS. Supplementary Table 1 lists NMR experimental details. Spectra were processed using TopSpin 3.1, with ^1^H frequencies referenced to DSS (0 ppm). Spectra were analyzed using CcpNmr Analysis 2.4.2^47^. Sequential assignment and all ^1^H chemical shifts (H_N_, H_α_, H_β_, H_γ_, H_δ_, and H_ε_) were assigned manually using ^1^H-^1^H TOCSY and NOESY spectra and used to identify the corresponding directly-bonded ^13^C (C’, C_α_, C_β_, C_γ_, C_δ_, and C_ε_) through ^1^H-^13^C HSQC cross-peaks. H_N_, H_α_, C_α_, and C_β_ assignments were compared to expected chemical shifts for random coil peptides in dimethyl sulfoxide (DMSO)^20^. Secondary chemical shifts (Δδ) for H_α_, C_α_, and C_β_ were used for CSI calculation^19,48^, using the optimized cut-offs for α-helices in the DMSO environment^20^. For comparison, the Dihedral Angles from Global Likelihood Estimates (DANGLE) algorithm was employed to predict secondary structuring^49^, as implemented in CcpNmr Analysis.

### Microscopy and Indirect Immunofluorescence

Confocal imaging of *S. pombe* containing GFP tagged *Sp*NHE1 was performed on an Olympus IX81 microscope equipped with a spinning-disk optimized by Quorum Technologies (Guelph, ON, Canada). Images were acquired using the software Volocity (Improvision Inc., Lexington, MA) with a 60× objective on a Hamamatsu EM-CCD camera (Hamamatsu, Japan). Yeast cells were either immobilized with 1% gelatin or for live cell imaging of the GFP tag, confocal microscopy was essentially as described earlier^23^.

## Electronic supplementary material

Supplementary Figures
