# Supplementary material for: Transmembrane Segment XI of the Na+/H+ Antiporter of S. pombe is a Critical Part of the Ion Translocation Pore
Source: Sci Rep. 2017 Oct 16;7:12793. doi: 10.1038/s41598-017-12701-z (PMC5643542; doi:10.1038/s41598-017-12701-z)
Supplement: Supplementary file 1 — Supplementary Figures [file 41598_2017_12701_MOESM1_ESM.pdf]

Supplementary Information for

Transmembrane Segment XI of the Na^+/H^+ Antiporter of *S. pombe* is a Critical Part of the Ion Translocation Pore

Debajyoti Dutta, Kyungsoo Shin, Jan K. Rainey and Larry Fliegel

Supplementary Fig. 1 A

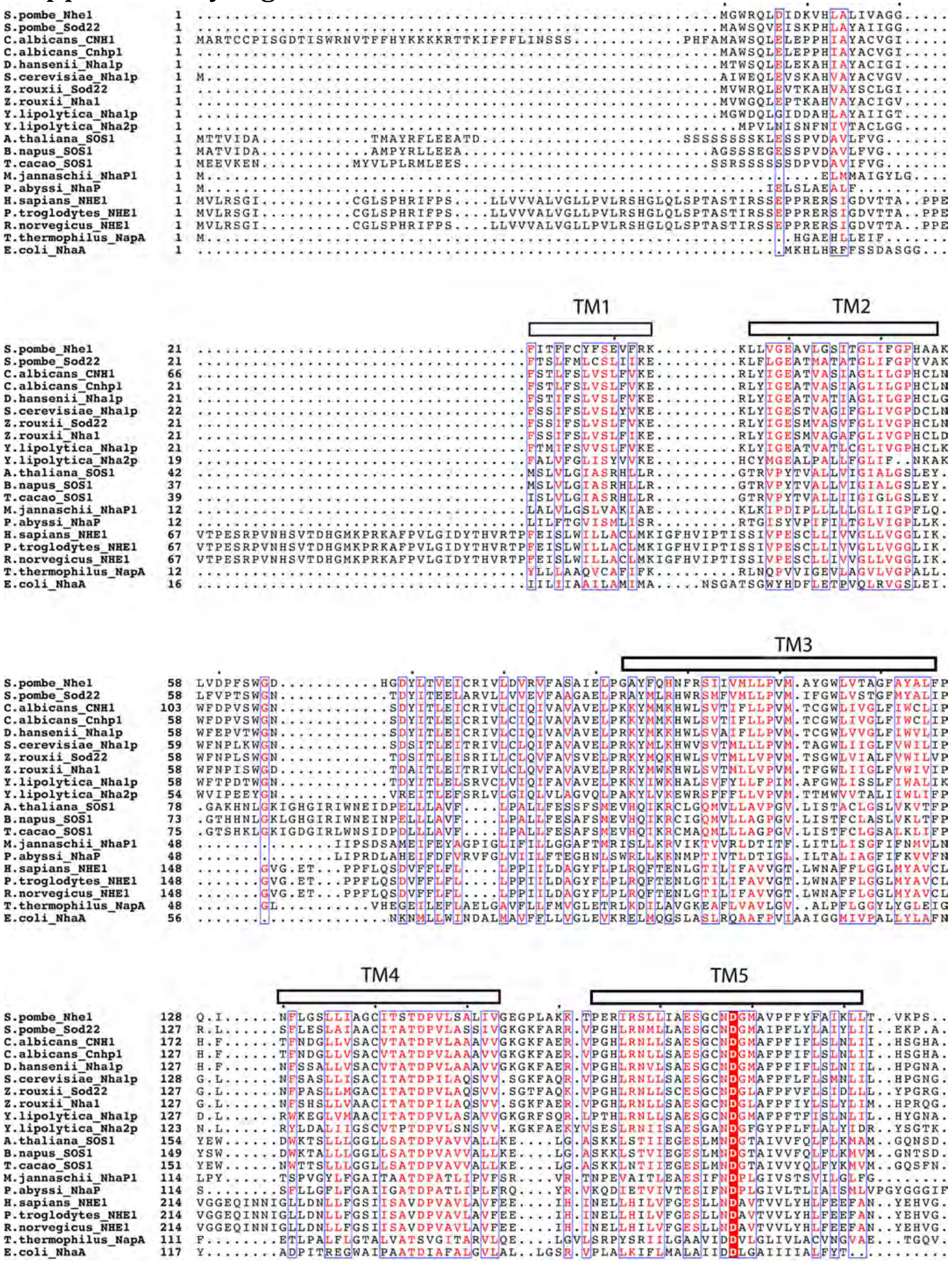

Supplementary Fig. 1 B

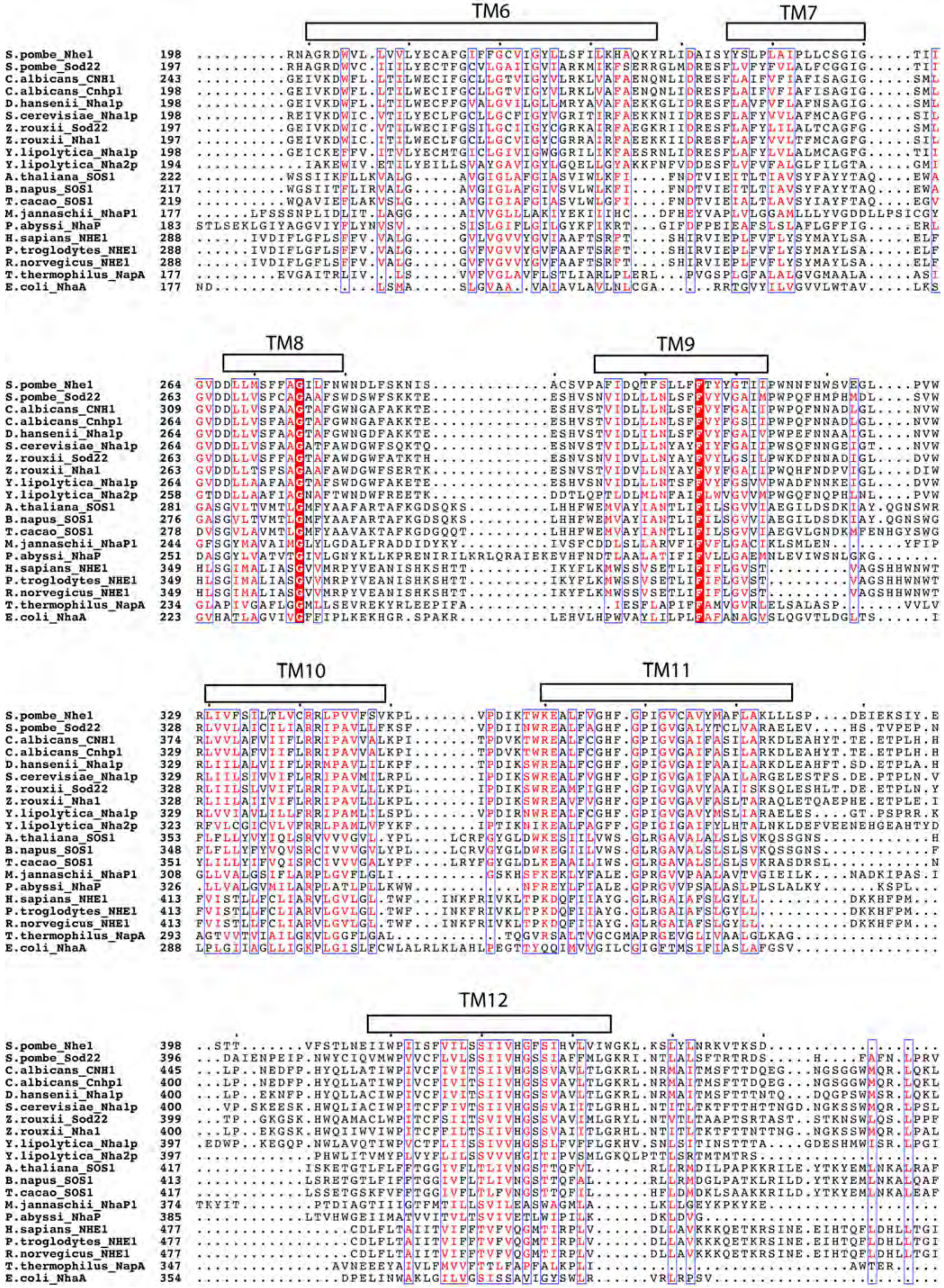

Supplementary Figure 1. **A,B,** Full alignment of sequences of yeast, fungi, plant, mammalian and bacterial plasma membrane Na^+/H^+ exchangers. See Fig. 1 for sequence sources. Predicted TM segments of *Sp*NHE1 are highlighted on the top of the sequence alignment. Conserved amino acids are colored red and conserved regions are boxed.

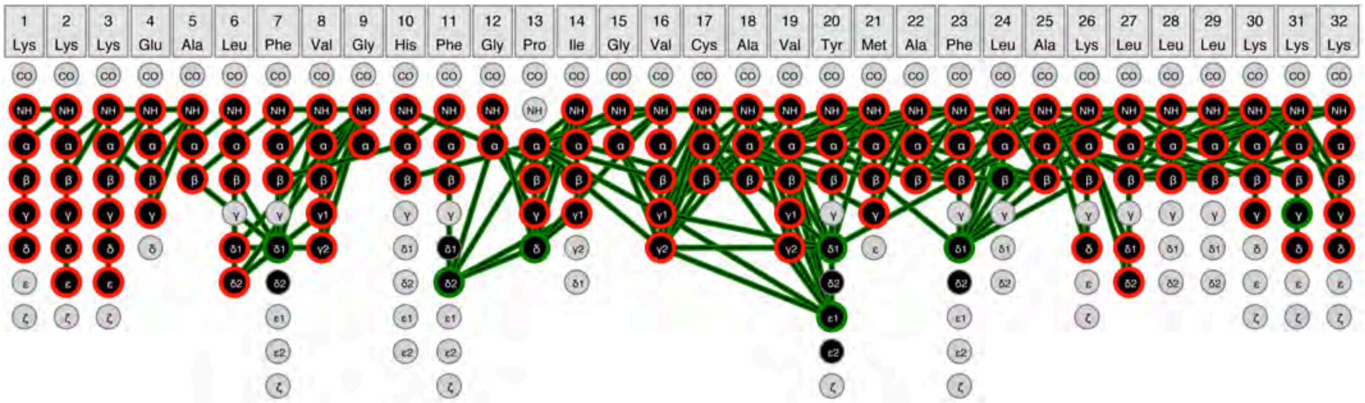

Supplementary Figure #2. Nucleus-by-nucleus plot of network of unambiguous ^{1}H-^{1}H homonuclear TOCSY (red) and NOESY (green) cross-peak assignments for TMXI peptide in DPC micelles (figure produced using CcpNmr Analysis). Good coverage of most side chains is observed, and extensive sequential and medium range NOE contacts are apparent, particularly through the C-terminal region of the peptide.

Supplementary Fig. 3. Growth of *S. pombe* containing either wild type or TM IV mutant *Sp*NHE1 proteins in liquid media with various concentrations of NaCl. To assess NaCl tolerance of strains media was inoculated with 2 X 10^6 cells into 2.5 ml of medium at 30 ^oC for up to 90 hours. Growth was monitored by measuring cell absorbance of suspensions at 600 nm at the indicated times. Results are the mean +/- SE of at least three determinations. *S. pombe* were grown in the presence of 0, 0.2 or 0.5 M NaCl as shown. **A-D** comparison of growth rates in NaCl medium of control, *Sod2::ura4 cells, S. pombe* containing *Sp*NHE1 proteins with alanine mutations. **E**, second round of mutagenesis to other amino acids. *Sod2:Ura4* refers to *S. pombe* with the *Sp*NHE1 knockout.

A

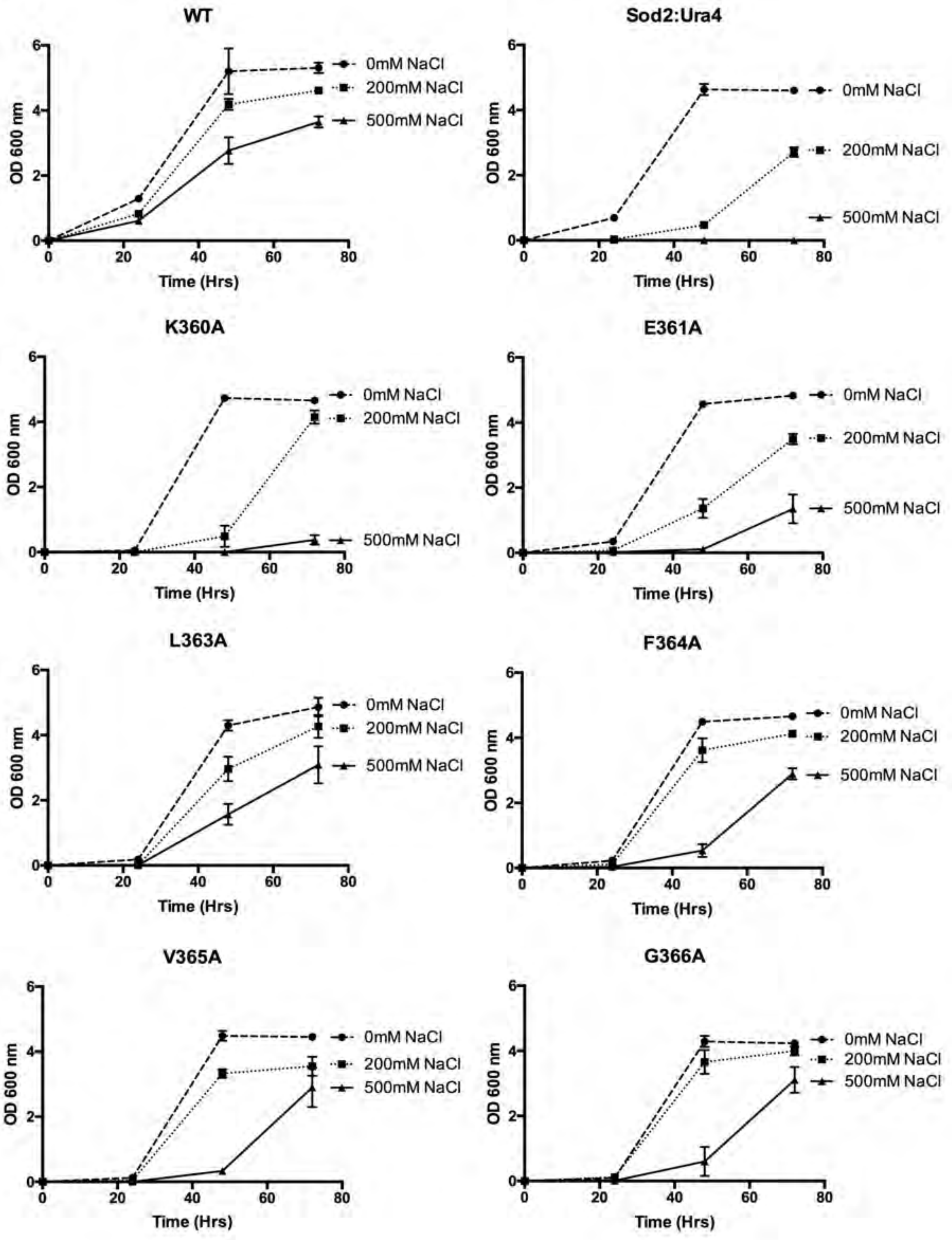
WT
Sod2:Ura4
K360A
E361A
L363A
F364A
V365A
G366A
OD 600 nm
Time (Hrs)
0mM NaCl
200mM NaCl
500mM NaCl

B

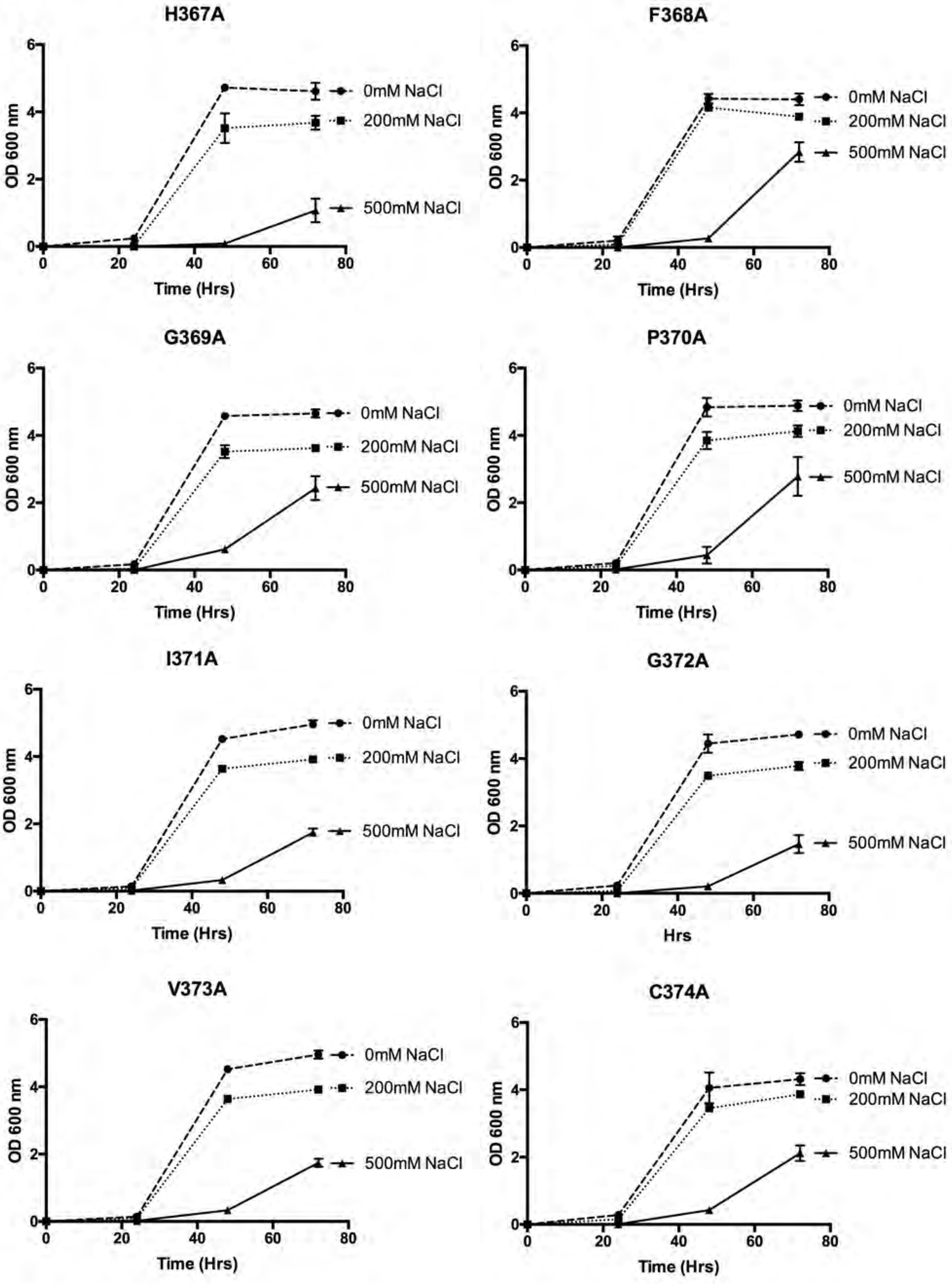
H367A
F368A
G369A
P370A
I371A
G372A
V373A
C374A
OD 600 nm
Time (Hrs)
Hrs
0mM NaCl
200mM NaCl
500mM NaCl

C

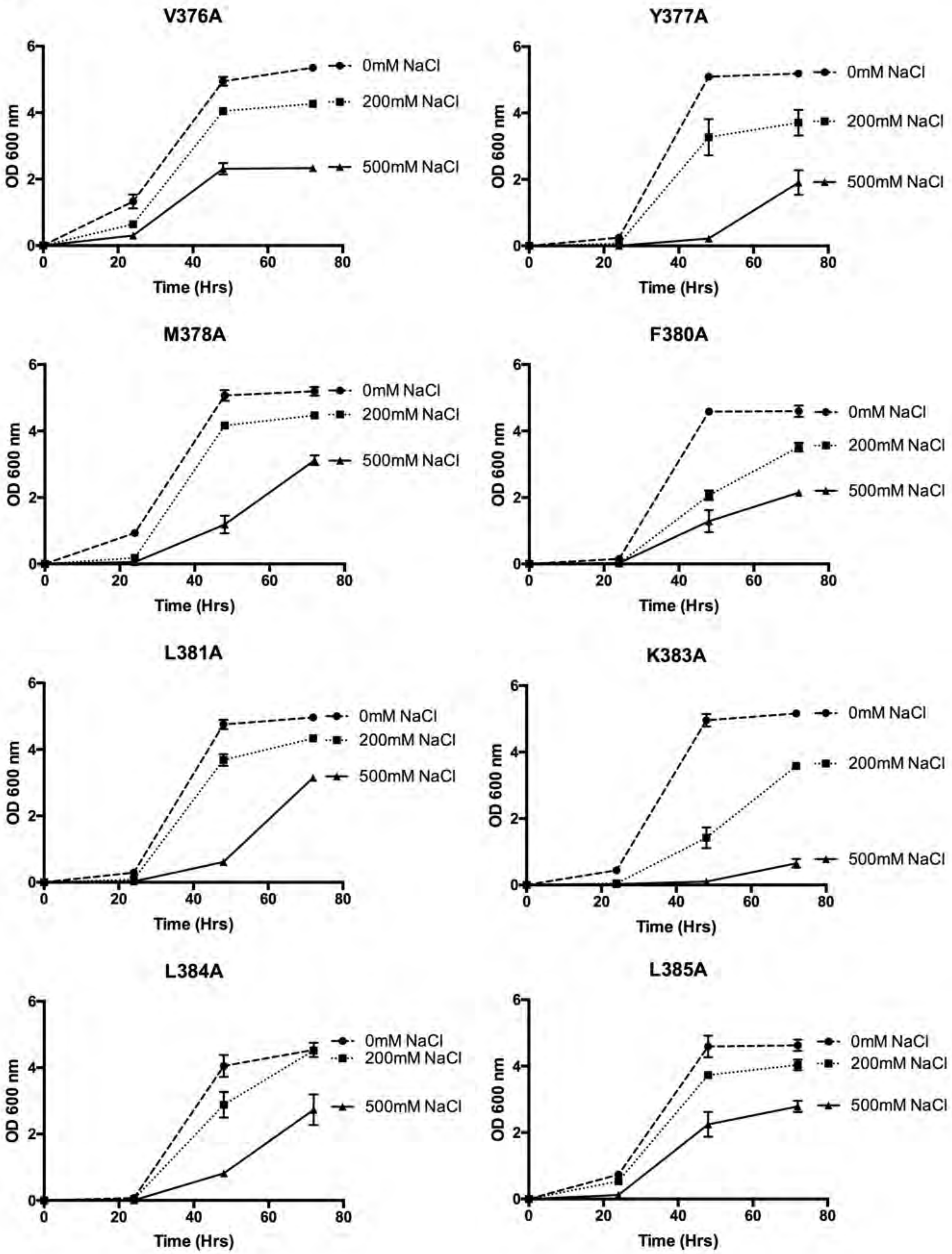
V376A
Y377A
M378A
F380A
L381A
K383A
L384A
L385A
OD 600 nm
Time (Hrs)
0mM NaCl
200mM NaCl
500mM NaCl
0
20
40
60
80
2
4
6

D

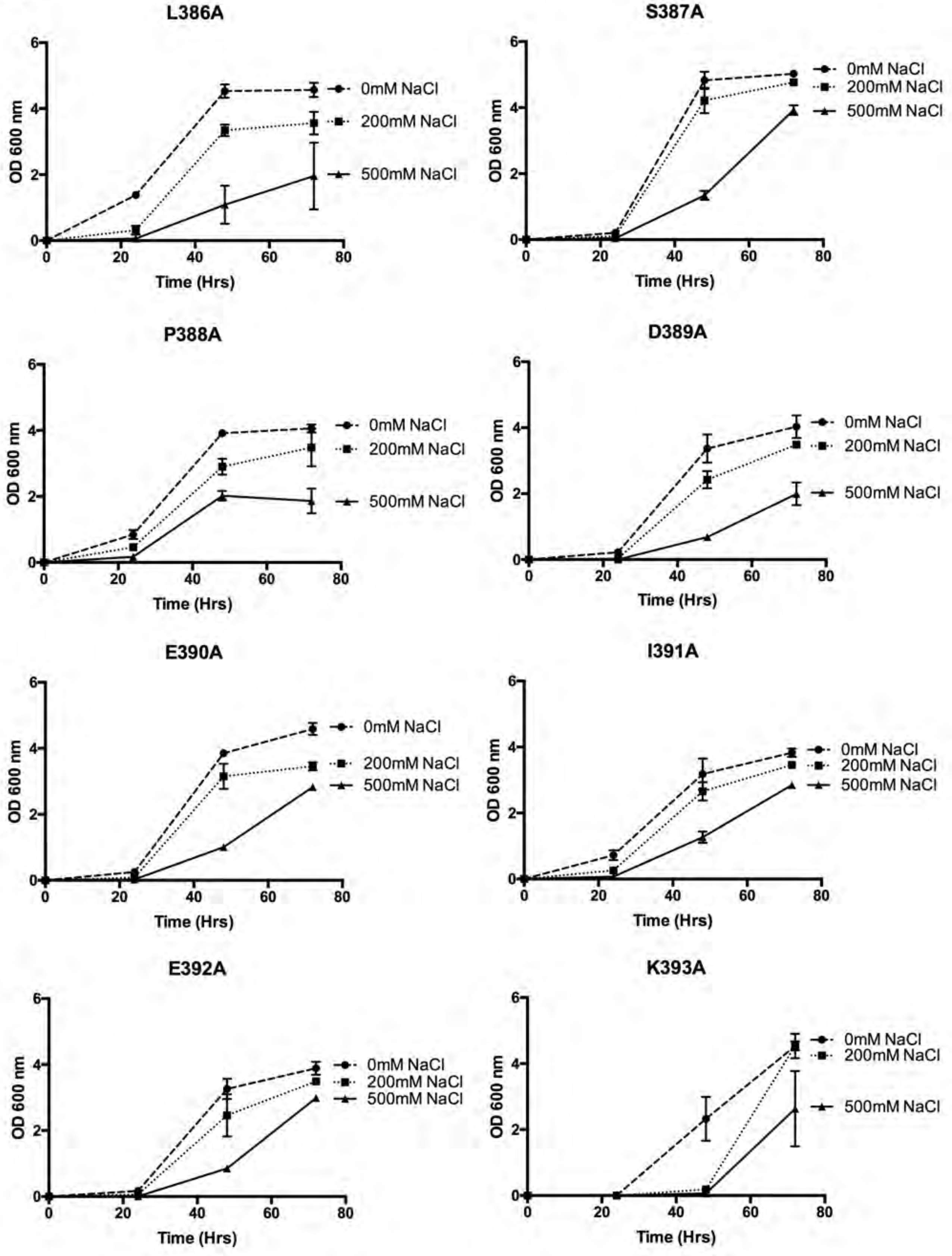
L386A
S387A
P388A
D389A
E390A
I391A
E392A
K393A
OD 600 nm
Time (Hrs)
0
20
40
60
80
2
4
6
0mM NaCl
200mM NaCl
500mM NaCl

E

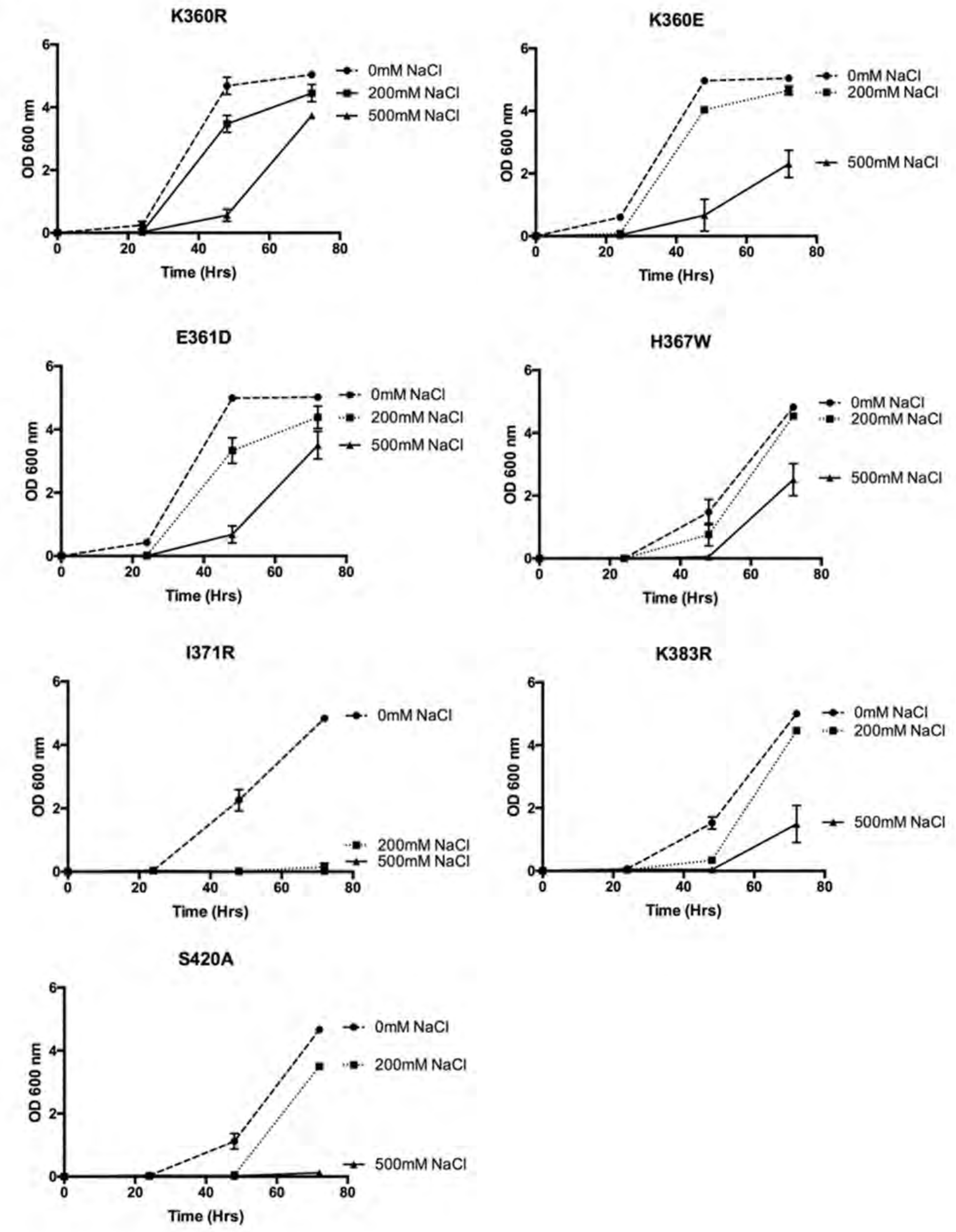
K360R
OD 600 nm
Time (Hrs)
0mM NaCl
200mM NaCl
500mM NaCl
K360E
OD 600 nm
Time (Hrs)
0mM NaCl
200mM NaCl
500mM NaCl
E361D
OD 600 nm
Time (Hrs)
0mM NaCl
200mM NaCl
500mM NaCl
H367W
OD 600 nm
Time (Hrs)
0mM NaCl
200mM NaCl
500mM NaCl
I371R
OD 600 nm
Time (Hrs)
0mM NaCl
200mM NaCl
500mM NaCl
K383R
OD 600 nm
Time (Hrs)
0mM NaCl
200mM NaCl
500mM NaCl
S420A
OD 600 nm
Time (Hrs)
0mM NaCl
200mM NaCl
500mM NaCl

Supplementary Fig. 4. Growth of *S. pombe* containing either wild type or TM IV mutant *Sp*NHE1 proteins in liquid media with various concentrations of LiCl. To assess LiCl tolerance of strains were grown as in Fig. 3. *S. pombe* were grown in the presence of 0, 1, 2, 3, 4 or 5 mM LiCl as shown. Results are the mean +/- SE of at least three determinations. **A-D** comparison of growth rates in LiCl medium of control, *Sod2::ura4 cells, S. pombe* containing *Sp*NHE1 proteins with alanine mutations. **E**, second round of mutagenesis to other amino acids. *Sod2:Ura4* refers to *S. pombe* with the *Sp*NHE1 knockout described earlier (9).

A

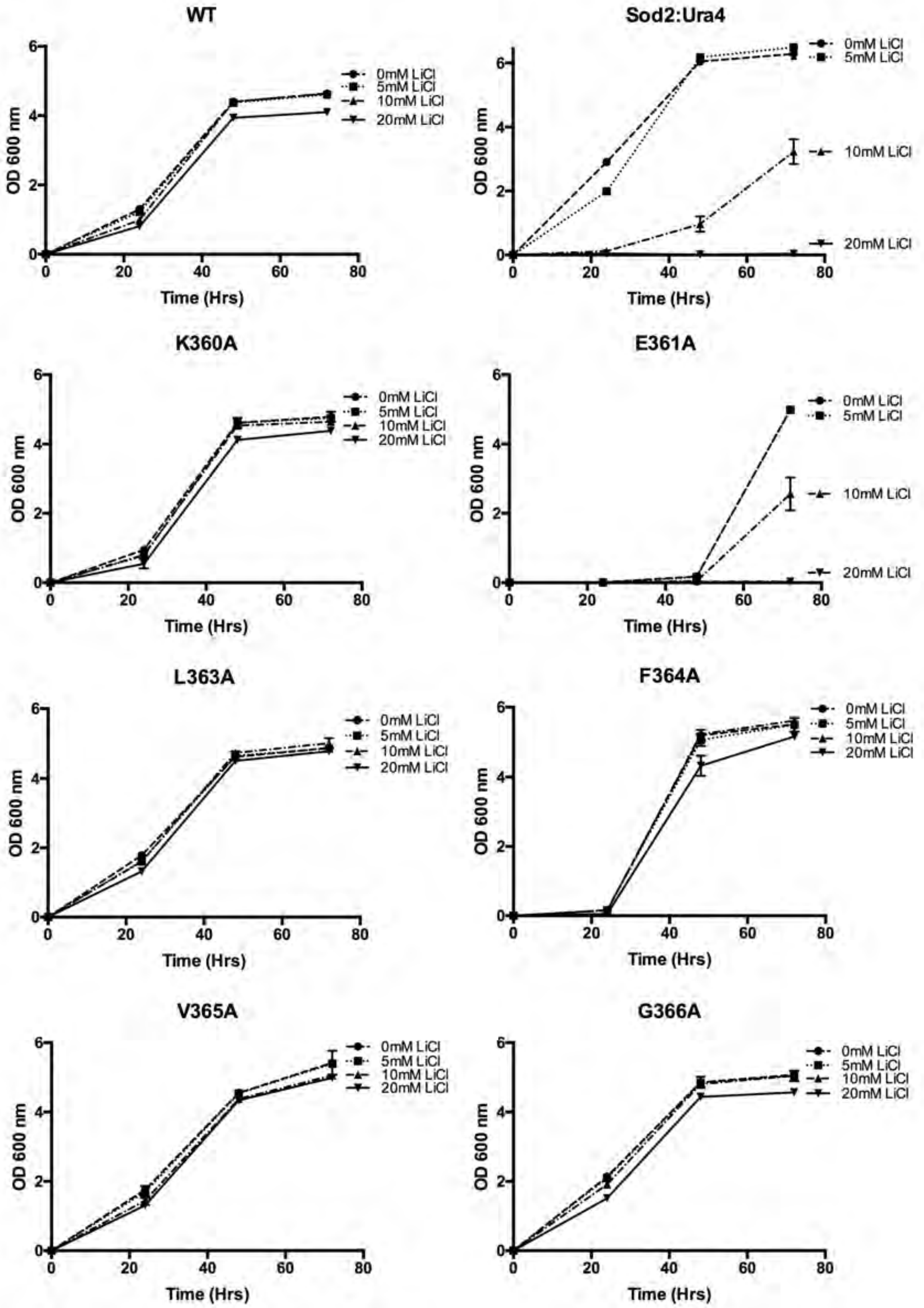

WT
Sod2:Ura4
K360A
E361A
L363A
F364A
V365A
G366A
OD 600 nm
Time (Hrs)
0mM LiCl
5mM LiCl
10mM LiCl
20mM LiCl

B

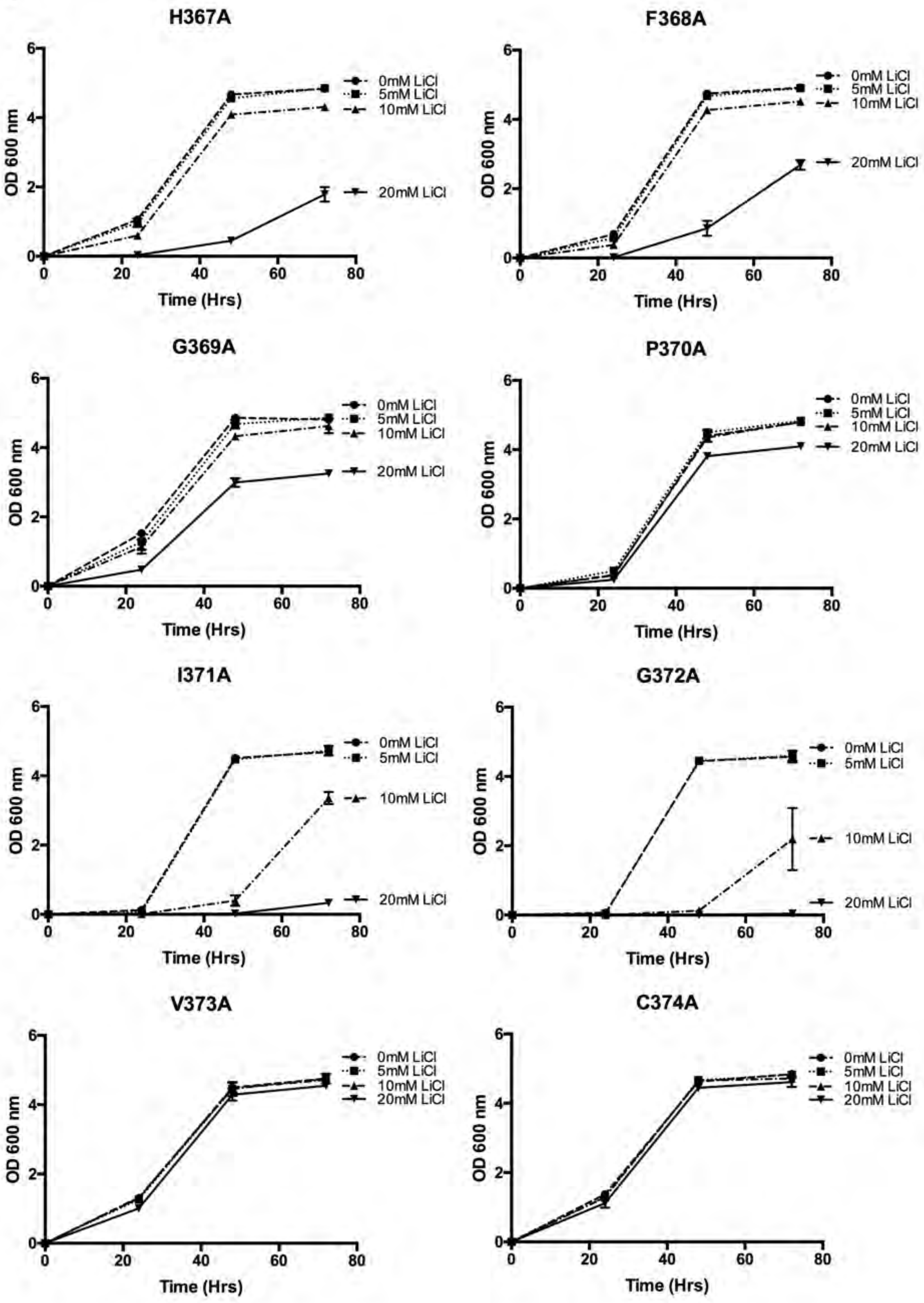
H367A
F368A
G369A
P370A
I371A
G372A
V373A
C374A
OD 600 nm
Time (Hrs)
0mM LiCl
5mM LiCl
10mM LiCl
20mM LiCl

C

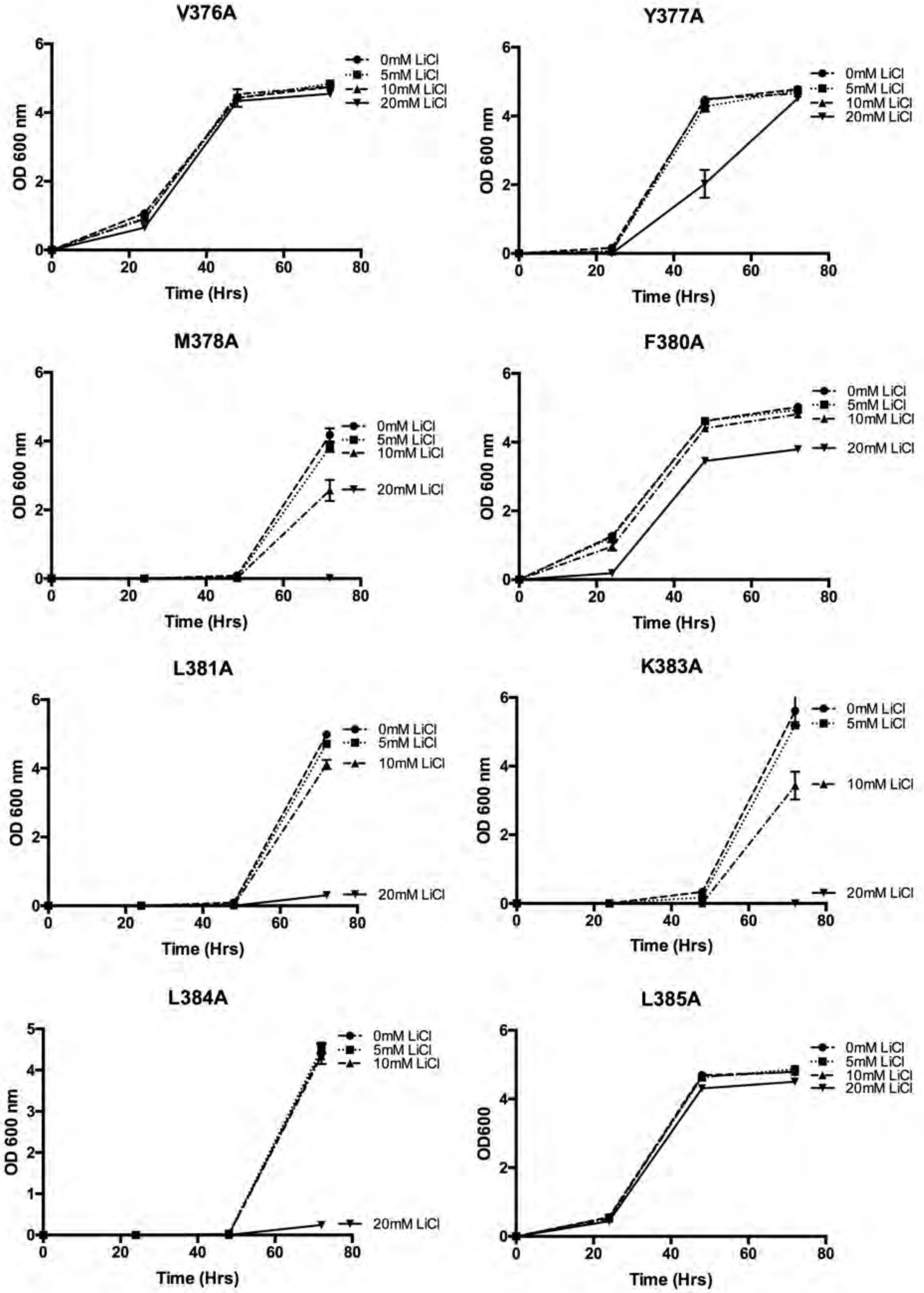
V376A
OD 600 nm
Time (Hrs)
0mM LiCl
5mM LiCl
10mM LiCl
20mM LiCl
Y377A
OD 600 nm
Time (Hrs)
0mM LiCl
5mM LiCl
10mM LiCl
20mM LiCl
M378A
OD 600 nm
Time (Hrs)
0mM LiCl
5mM LiCl
10mM LiCl
20mM LiCl
F380A
OD 600 nm
Time (Hrs)
0mM LiCl
5mM LiCl
10mM LiCl
20mM LiCl
L381A
OD 600 nm
Time (Hrs)
0mM LiCl
5mM LiCl
10mM LiCl
20mM LiCl
K383A
OD 600 nm
Time (Hrs)
0mM LiCl
5mM LiCl
10mM LiCl
20mM LiCl
L384A
OD 600 nm
Time (Hrs)
0mM LiCl
5mM LiCl
10mM LiCl
20mM LiCl
L385A
OD600
Time (Hrs)
0mM LiCl
5mM LiCl
10mM LiCl
20mM LiCl

D

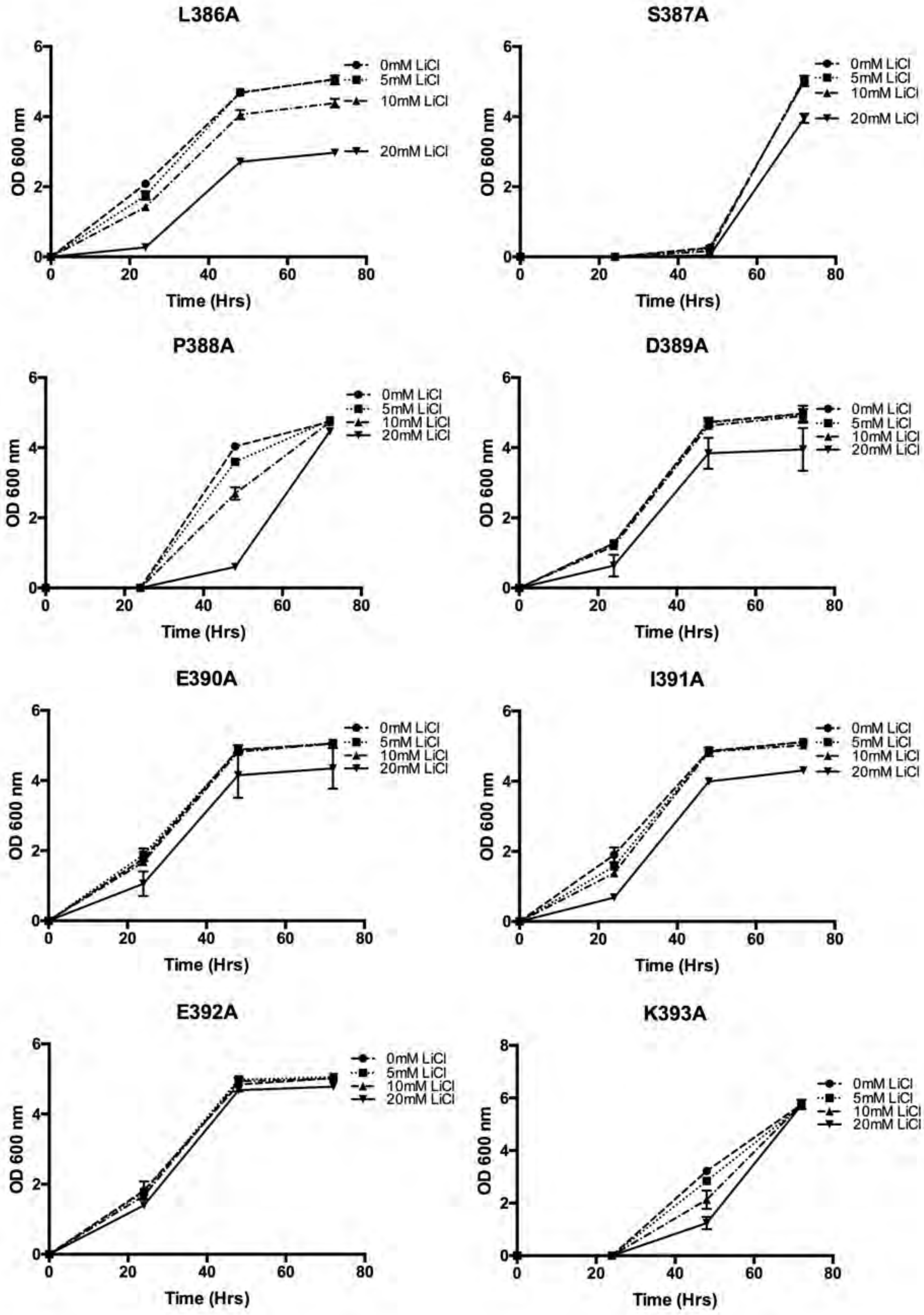
L386A
S387A
P388A
D389A
E390A
I391A
E392A
K393A
OD 600 nm
Time (Hrs)
0mM LiCl
5mM LiCl
10mM LiCl
20mM LiCl

E

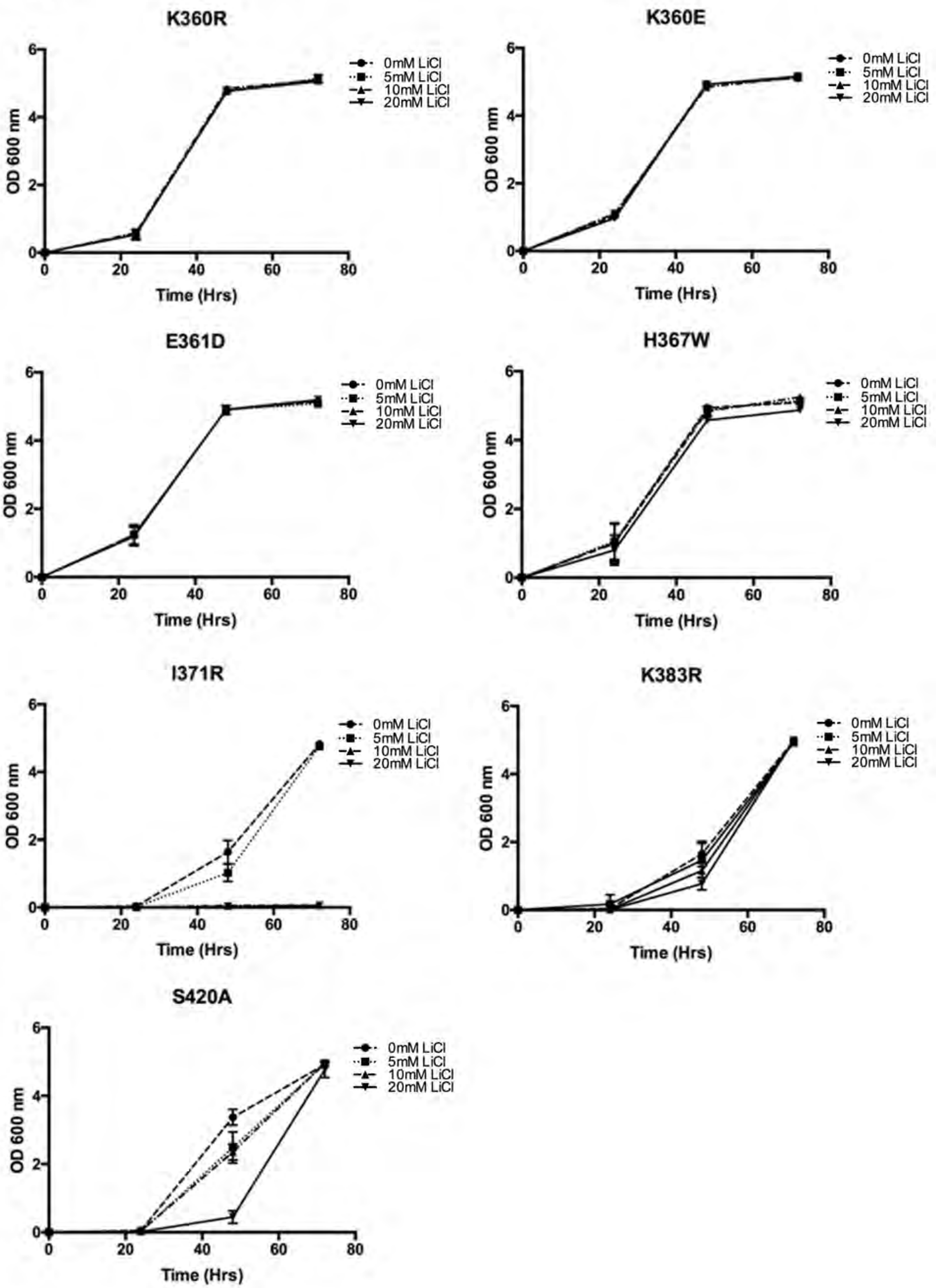
K360R
OD 600 nm
Time (Hrs)
0mM LiCl
5mM LiCl
10mM LiCl
20mM LiCl
K360E
OD 600 nm
Time (Hrs)
0mM LiCl
5mM LiCl
10mM LiCl
20mM LiCl
E361D
OD 600 nm
Time (Hrs)
0mM LiCl
5mM LiCl
10mM LiCl
20mM LiCl
H367W
OD 600 nm
Time (Hrs)
0mM LiCl
5mM LiCl
10mM LiCl
20mM LiCl
I371R
OD 600 nm
Time (Hrs)
0mM LiCl
5mM LiCl
10mM LiCl
20mM LiCl
K383R
OD 600 nm
Time (Hrs)
0mM LiCl
5mM LiCl
10mM LiCl
20mM LiCl
S420A
OD 600 nm
Time (Hrs)
0mM LiCl
5mM LiCl
10mM LiCl
20mM LiCl

Table 1: NMR experimental details

Experiment	Bruker pulse program	Number of scans	Relaxation delays (s)	Sweepwidth (ppm)	Transmitter frequency offset (ppm)	Acquisition time (s)	Acquired points and increments
^{13}C-^{1}H HSQC	hsqcetgpsp.2	128	1	^{13}C: 70<br>^{1}H: 14	^{13}C: 40<br>^{1}H: 4.705	^{13}C: 0.0000406<br>^{1}H: 0.1044480	2048 x 256
^{1}H-^{1}H TOCSY (60 ms mixing time)	dipsi2esgpph	32	1.5	^{1}H: 10.2018<br>^{1}H: 10.2016	^{1}H: 4.705<br>^{1}H: 4.705	^{1}H: 0.1433600<br>^{1}H: 0.0537614	2048 x 768
^{1}H-^{1}H NOESY (150, 200, 250 ms mixing time)	noesyesgpph	32	1.5	^{1}H: 10.2018<br>^{1}H: 10.2016	^{1}H: 4.705<br>^{1}H: 4.705	^{1}H: 0.1433600<br>^{1}H: 0.0537614	2048 x 768
